# Prominent positioning and food swaps are effective interventions to reduce the saturated fat content of the shopping basket in an experimental online supermarket: a randomized controlled trial

**DOI:** 10.1186/s12966-019-0810-9

**Published:** 2019-06-07

**Authors:** Dimitrios A. Koutoukidis, Susan A. Jebb, José M. Ordóñez-Mena, Michaela Noreik, Melina Tsiountsioura, Sarah Kennedy, Sarah Payne-Riches, Paul Aveyard, Carmen Piernas

**Affiliations:** 1 Nuffield Department of Primary Care Health Sciences, University of Oxford, Oxford, UK; 2 NIHR Oxford Biomedical Research Centre, Oxford, UK

**Keywords:** Saturated fat, Swaps, Default order, Online shopping, Supermarket, Food purchases, Diet, Randomized controlled trial

## Abstract

**Background:**

Interventions to reduce the saturated fat (SFA) content of food purchases may help reduce SFA consumption and lower cardiovascular risk. This factorial RCT aimed to examine the effect of altering the default order of foods and being offered a swap on the SFA content of food selected during an online shopping experiment.

**Methods:**

UK adults who were the primary grocery shoppers for their household were recruited online and invited to select items in a custom-made experimental online supermarket using a 10-item shopping list. Participants were randomly allocated to one of four groups (i) to see products within a category ranked in ascending order of SFA content, (ii) receive an offer to swap to a product with less SFA, (iii) a combination of both interventions, or (iv) no intervention. The primary outcome was the difference in percentage energy from SFA in the shopping basket between any of the four groups. The outcome assessors and statistician were blinded to intervention allocation.

**Results:**

Between March and July 2018, 1240 participants were evenly randomised and 1088 who completed the task were analysed (88%). Participants were 65% female and aged 38y (SD 12). Compared with no intervention (*n* = 275) where the percentage energy from SFA was 25.7% (SD 5.6%), altering the order of foods (*n* = 261) reduced SFA by [mean difference (95%CI)] -5.0% (− 6.3 to − 3.6) and offering swaps (*n* = 279) by − 2.0% (− 3.3 to − 0.6). The combined intervention (*n* = 273) was significantly more effective than swaps alone (− 3.4% (− 4.7 to − 2.1)) but not different than altering the order alone (− 0.4% (− 1.8 to 0.9)), *p* = 0.04 for interaction.

**Conclusions:**

Altering the default order to show foods in ascending order of SFA and offering a swap with lower SFA reduced percentage energy from SFA in an experimental online supermarket. Environmental-level interventions, such as altering the default order, may be a more promising way to improve food purchasing than individual-level ones, such as offering swaps.

**Trial registration:**

ISRCTN13729526 10.1186/ISRCTN13729526 26th February 2018.

**Electronic supplementary material:**

The online version of this article (10.1186/s12966-019-0810-9) contains supplementary material, which is available to authorized users.

## Introduction

Reducing the dietary intake of saturated fat (SFA) can lower low-density lipoprotein cholesterol and reduce cardiovascular disease risk [1, 2]. The recent WHO draft guidelines recommend reducing the intake of SFA to less than 10% of total energy intake [3]. However, average SFA intake in the UK (13.5% of energy intake) remains more than a third higher than recommended [4]. Similar high SFA intakes are observed in the USA and other high- and middle- income countries [5, 6], and progress in reducing SFA through public education programs has been slow. Hence, novel approaches are needed to achieve this target at the population level.

Food purchasing is a key determinant of food consumption and interventions targeting the nutritional quality of food during shopping present a clear opportunity for an intervention with wide reach. Individual-level interventions previously identified in systematic reviews as effective behaviour change techniques (e.g. tailored dietary advice, information, self-monitoring and personalised feedback) can be easily applied in the context of online supermarket shopping [7, 8]. A previous study recommending lower SFA options at the point of purchase showed a significant reduction in total SFA from online food purchases with no difference in expenditure [9].

Individual-level interventions require reflection and analytical decision-making, but, in practice, many decisions about food are not reflective, conscious choices but are automatic reactions prompted by environmental cues [10]. Given this, there is also growing interest in environmental-level interventions to change eating behaviours by altering the defaults at the point of choice, so called choice architecture or nudging interventions [11]. Preliminary evidence suggests that these environmental-level interventions might achieve a meaningful impact on behaviours and could be applied in the retail environment to influence food purchasing [7]. Given that both types of interventions have been shown to be effective, we hypothesised that their combination will lead to larger effects sizes.

The aim of this trial was to test the effectiveness of an environmental-level intervention (i.e. altering the default order of foods to show foods in ascending order of SFA) and an individual-level intervention (i.e. an explicit offer to swap to an alternative food with lower SFA) on the SFA content of online food shopping. In this proof-of concept trial, we used an experimental online research shopping platform to explore the effectiveness of these interventions, alone or in combination, compared to no intervention.

## Subjects and methods

### Design and setting

This was a prospectively registered 2 × 2 factorial randomised controlled trial conducted in a custom-made simulated online supermarket platform (www.woodssupermarket.co.uk) developed by Cauldron, UK (http://cauldron.sc/clients#woods). The supermarket was developed to emulate a real online supermarket website as previously described [12]. It contains a food database with ~ 11,000 products, downloaded from a real UK grocery retailer (Tesco.com API, February 2012), which includes standard UK branded products. Nutrient composition information per 100 g was supplemented by manual linkages with food labels at online supermarket websites and with data provided by Kantar WorldPanel and the Medical Research Council Human Nutrition Research food and nutrient database [13]. Data were collected and managed using the supermarket platform and the REDCap (Research Electronic Data Capture) electronic data capture tools hosted at the University of Oxford [14]. REDCap is a secure, web-based application designed to support data capture for research studies, providing: 1) an intuitive interface for validated data entry; 2) audit trails for tracking data manipulation and export procedures; 3) automated export procedures for seamless data downloads to common statistical packages; and 4) procedures for importing data from external sources. The protocol was implemented without changes except minor prospective revisions, made prior to analysis which are noted in the analysis plan (Additional file 1).

### Participants

Participants were recruited between March and July 2018 through an online research agency [15]. Invitations were sent to a random subsample of a pool of 6968 who had all been pre-screened as eligible. Due to a technical limitation, the invitation and response rates were not recorded. Participants were eligible if they lived in the UK, were aged 18 years or over, were the main (or shared) grocery shopper for their household, were able to read English, had access to a computer and Internet connection (by virtue of being part of the Prolific participant pool), and were willing and able to provide informed consent. People were not eligible if they were following any restricted diet such as a vegetarian, vegan, dairy-free, sugar-free, or gluten-free diet. Following online screening for these criteria, participants provided consent electronically. Following consent, they answered standard demographic questions as well as a few additional questions on shopping habits and health status at baseline (Additional file 1: Appendix B).

### Randomization

The statistician generated the randomization sequence using the R package ‘blockrand’ [16] and the lead researcher uploaded the sequence in REDCap. Following the baseline questionnaire, participants were allocated to trial groups by REDCap via computerised random number generation on a 1:1:1:1 basis with random block sizes. Allocation concealment was achieved, as participants were recruited from Prolific independently of the research team and automatically randomised without human involvement.

### Shopping task

Following randomization, participants were redirected to the supermarket website that introduced the shopping task. The website explained how to complete the task. As with real online supermarkets, participants could find items by browsing the supermarket departments and shelves or using a search function. They were asked to select 10 ‘everyday’ foods from a pre-specified shopping list. They were instructed to imagine they were doing their own grocery shopping and to choose foods that they and their household would want to eat. The 10 foods were major sources of SFA in the UK, within food categories where lower SFA options are also available. Participants were not prevented from selecting as many items as they wished, in unlimited quantities, but the instructions requested selection of only a single item per category from the shopping list below. The list comprised:

- Milk for everyday use
- Butter or margarine for everyday use
- Cheese for use in sandwich or light meal
- Ready-to-eat savoury entree item (e.g. cured meats, samosas)
- Ready-to-eat individual chilled dessert
- Meat/fish/vegetarian alternative to cook for 4 people
- Dessert for a meal of 4 people
- Something to eat with a hot drink
- A sweet snack item to eat now
- A savoury snack item to eat now

In the consent process, all participants were informed in one sentence that: “This study aims to investigate if two different ways of making healthier choices when shopping online are acceptable to shoppers and effective in reducing the saturated fat in the foods in their basket.” However, this sentence was among many in the participant information pages and no further reference was made to SFA afterwards except in the swap condition as a necessary part of that intervention (see below). Following completion of this task, participants were redirected to REDCap to complete a survey assessing the intervention acceptability, their usual shopping behaviour, and an open-ended box for comments. Upon completion, participants were reimbursed with £5 for their participation.

### Interventions

Participants were randomly allocated to one of the following groups:

#### Swaps (individual-level intervention)

**Fig. 1:**
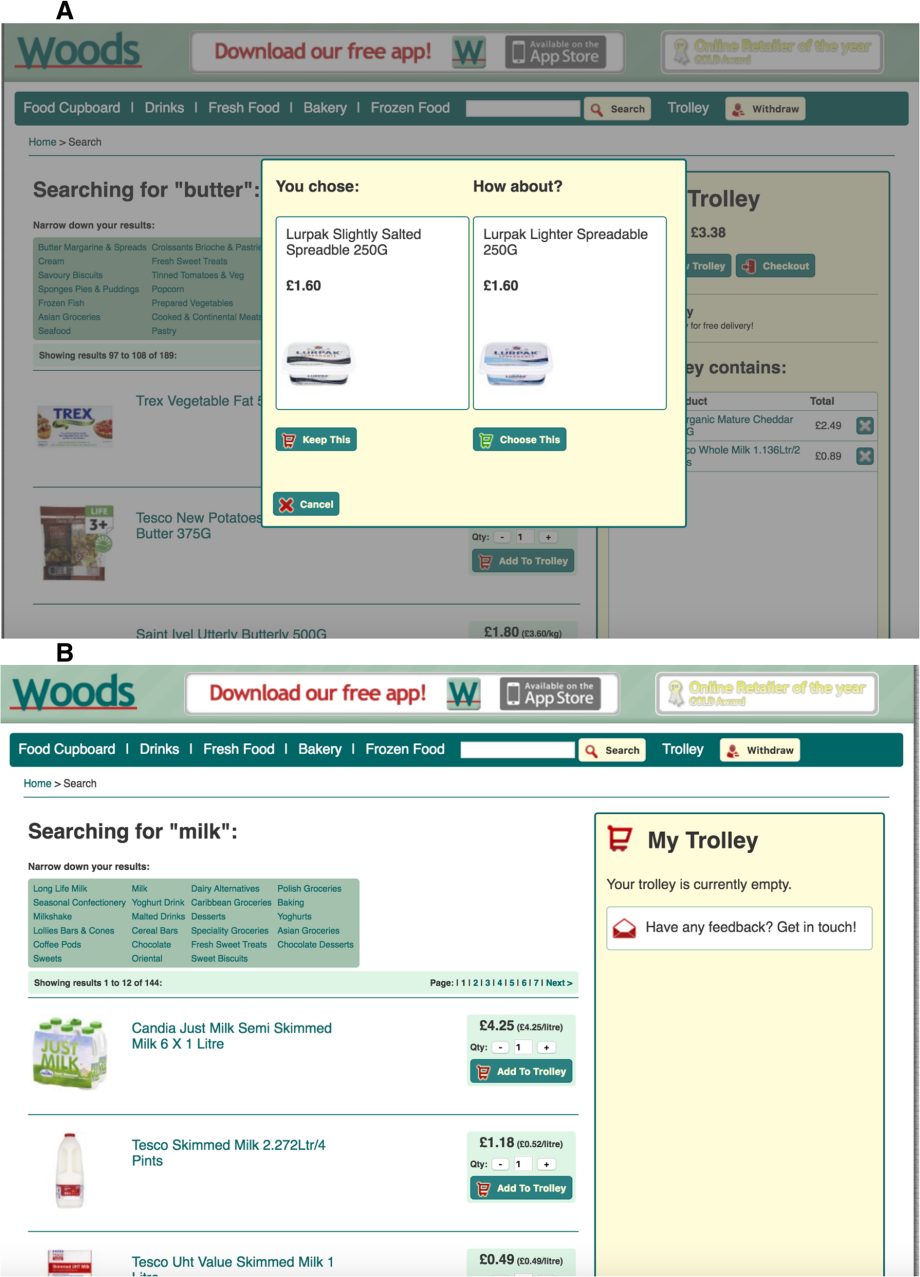
An example of the individual-level intervention when selecting butter whereby an explicit swap is offered matched for category, weight, price, and brand (**a**) and an example of the environmental-level intervention when searching for milk whereby products are shown in ascending order of saturated fat content (**b**)

Participants were offered an explicit swap with less SFA. Swaps were offered if an alternative product within the same category had at least 2 percentage points less SFA (i.e. 2 g less SFA per 100 g of product), was between 60 and 140% of the weight of the original product, and was between 0 and 200% of the price of the original product. Swaps were offered at the point of selection, immediately after selecting an item to be added to their shopping basket. If multiple swaps were available, the one of the same brand as the base product was offered and if no products of the same brand were available a swap that met the criteria was randomly offered. An example swap is shown in Fig. 1a. Before commencing the task, participants in this group were advised that they might be offered a swap with lower SFA. They were advised to choose the food with lower SFA only if they would choose this food if offered in their normal shop, choose it if they and their family would eat it gladly, or are prepared to eat it to lower their SFA intake. They were advised not to choose the product if they wouldn’t be willing to eat it. If they refused the swap, no further swaps for that food were offered.

#### Altering the default order (environmental-level intervention)

When searching or browsing foods, participants viewed a list of products in ascending order of SFA content (i.e. the products with the lowest SFA content appeared at the top of the screen) but this order was not made explicit to participants. Moreover, the SFA content of the food was not displayed in the product list but, in common with all UK online supermarkets, the SFA content along with other nutrients from the nutrient facts panel was shown if the participant clicked on a product in search of more information. The SFA order was applied to each list of foods offered to participants when searching for products. An example of the intervention is shown in Fig. 1b.

#### Combination of individual- and environmental-level interventions

Participants allocated to this arm received both interventions as described above. Participants were exposed to the environmental-level intervention while searching for items and the individual-level intervention after selecting specific items. Accordingly, the environmental-level intervention was viewed before the individual-level one.

#### Control

Participants in this arm shopped using the default version of the website with a random order of the foods displayed in response to searching or browsing with no swaps offered.

### Blinding

Investigators were not blinded to intervention allocation, but they were not able to manipulate any study parameter following the initial study set up, as all study procedures took place in the online platform. The outcome assessment was blinded, as it happened automatically in the online platform. The statistician was blinded to intervention allocation. Participants were necessarily unblinded and were aware of the study aims.

### Primary outcome

The primary outcome was the difference in the SFA content of the basket measured as the difference in the percentage of total energy between each of the four trial groups. We adjusted for total energy, because it places the focus on the nutritional composition of the foods selected and not the total amount of food purchased, as it would be the case if we used an absolute amount of saturated fat. Furthermore, it makes the outcome comparable to the nutritional recommendations for saturated fat, which are expressed as percentage of energy.

### Secondary outcomes

We examined the differences between the baskets in each group:

i. the proportion of products with less than 1.5 g of SFA per 100 g of product (1.5% SFA) [17]
ii. cost of the basket, expressed as £/100 g
iii. total energy (kcal), energy density (kcal/100 g), sugar (percentage of total energy), and salt (g/100 g).

We also examined the differences between offering swaps alone and the combined intervention arm in:

i. the percentage of energy from SFA per swap accepted
ii. the proportion of swaps accepted out of those offered
iii. the proportion of swaps accepted out of those offered by median observed change in SFA (high SFA change vs low SFA change)
iv. the proportion of swaps accepted out of those offered for (a) butter, margarine, and spreads, (b) cheese, (c) milk, (d) meat and meat products, and (e) sweets and desserts, including chocolates, sweets, ice cream, cakes, pies, biscuits, and sweet items from the bakery
v. the proportion of accepted swaps out of the number of items selected.

### Sample size

The relationship between SFA intake and cardiovascular outcomes is linear and so at a population-level even very small reductions in SFA intake will be of public health significance. We made a pragmatic decision to power this study based on a 2% reduction in energy from SFA. This magnitude of reduction is estimated to be associated with an 11% lower risk of cardiovascular disease mortality [18]. Assuming 7% standard deviation in the total basket between any of the 4 groups and using intention to treat analyses with 90% power and two-sided α = 0.05, we required 258 participants per group (total *n* = 1032). A final sample of 1240 participants would account for 20% attrition of participants not completing the shopping task.

### Statistical analysis

We followed a pre-specified statistical plan (Additional file 1) published in advance of the analysis in the ISRCTN registry (ISRCTN13729526). An independent trial statistician analysed the primary outcome and secondary outcomes i-iii using two-way analysis of variance. As the comparisons had been pre-specified, we did not correct for multiple testing [19]. We also tested for interaction between the two interventions and the main outcome by introducing an interaction term in the regression model [20]. Secondary outcomes iv-viii are presented as medians with interquartile range (IQR). We analysed data from participants who bought at least one product from at least 5 out of 10 categories of the shopping list and, when participants bought more than the 10 items requested, we included all items bought. We performed pre-specified subgroup analyses by sex, age (below or above the median), ethnic group (white vs non-white), obesity, education (none/secondary vs higher), and household income (low/middle vs higher). Estimates of comparative effectiveness for all outcomes are reported as mean differences with 95% confidence intervals (CI). Two researchers analysed the open-ended comments using manifest content analysis counting the frequency and grouping specific content evident in the comments [21]. All statistical analyses were conducted in R (version 3.5.0, Vienna, Austria).

## Results

**Fig. 2:**
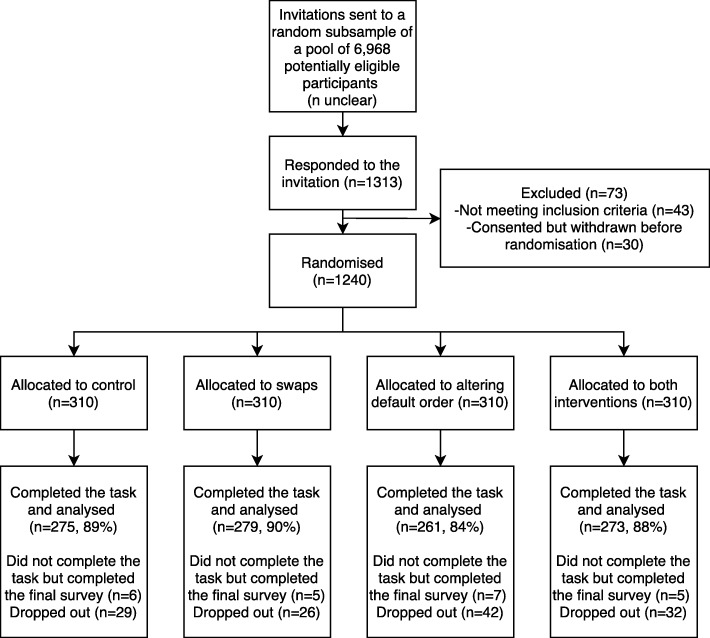
CONSORT flow diagram

Out of the 6968 eligible participants, 1240 consented and were evenly randomised to the four groups. Data from 1088 (88%) who completed the task were analysed (Fig. 2). Participants were on average (SD) 38 (12) years old, two thirds were female, a third were affected by obesity (BMI ≥ 30 kg/m^2^), 90% were of white background and about three quarters had shopped online for groceries at least once in the last year (Table 1).

**Table 1:** Baseline characteristics of the trial participants

	Control		Swaps		Altering order		Combined	
	(*n* = 310)		(*n* = 310)		(*n* = 310)		(*n* = 310)	
Age, *years*, mean ± SD	37.5 ± 11.7		37.8 ± 12.9		38.2 ± 12.7		37.2 ± 11.7	
Sex, *female*, *n* (%)	193 ± 62.3		207 ± 66.8		214 ± 69		203 ± 65.5	
BMI, *kg/m*^*2*^, mean ± SD	27.8 ± 7.2		27.9 ± 7.3		27.8 ± 6.4		27.4 ± 6.3	
BMI category, *n* (%)								
*< 18.5*	5	(1.6)	5	(1.6)	7	(2.3)	7	(2.3)
*18.5–24.9*	113	(36.5)	130	(41.9)	113	(36.5)	123	(39.7)
*25–29.9*	101	(32.6)	79	(25.5)	89	(28.7)	90	(29)
*> 30*	87	(28.1)	95	(30.6)	96	(31)	86	(27.7)
*Missing*	4	(1.3)	1	(0.3)	5	(1.6)	4	(1.3)
Ethnic group, *n* (%)								
*White*	272	(87.7)	285	(91.9)	285	(91.9)	280	(90.3)
*Asian/Black*	20	(6.5)	10	(3.2)	13	(4.2)	16	(5.2)
*Mixed/Other*	18	(5.8)	14	(4.5)	11	(3.5)	13	(4.2)
*Missing*	0	(0)	1	(0.3)	1	(0.3)	1	(0.3)
Education, *n* (%)								
*None*	7	(2.3)	11	(3.5)	17	(5.5)	10	(3.2)
*Secondary*	74	(23.9)	105	(33.9)	108	(34.8)	86	(27.7)
*Higher*	229	(73.9)	193	(62.3)	184	(59.4)	213	(68.7)
*Missing*	0	(0)	1	(0.3)	1	(0.3)	1	(0.3)
Household income, *n* (%)								
*Lower (£25 k)*	124	(40)	142	(45.8)	135	(43.5)	125	(40.3)
*Middle (£26-39 k)*	93	(30)	70	(22.6)	77	(24.8)	79	(25.5)
*Higher (£40 k)*	83	(26.8)	90	(29)	83	(26.8)	95	(30.6)
*Missing*	10	(3.2)	8	(2.6)	15	(4.8)	11	(3.5)
Household size, median (IQR)	3.0 (2–4)		3.0 (2–4)		3.0 (2–4)		3.0 (2–4)	
Household average supermarket expenditure, *£*, median (IQR)	70.0 (50–100)		60.0 (45–90)		60.0 (50–85)		60.0 (50–100)	
Online grocery shopping, *n* (%)								
*> =1/week*	21	(6.8)	18	(5.8)	27	(8.7)	32	(10.3)
*1–3 times/month*	42	(13.5)	38	(12.3)	54	(17.4)	62	(20)
*4–11 times in the last year*	78	(25.2)	76	(24.5)	79	(25.5)	74	(23.9)
*1–3 times in the last year*	91	(29.4)	91	(29.4)	71	(22.9)	72	(23.2)
*Never or not in the last year*	78	(25.2)	87	(28.1)	78	(25.2)	69	(22.3)
*Missing*	0	(0)	0	(0)	1	(0.3)	1	(0.3)
Online non-grocery shopping, *n* (%)								
*> =1/week*	35	(11.3)	43	(13.9)	27	(8.7)	33	(10.6)
*1–3 times/month*	137	(44.2)	124	(40)	129	(41.6)	133	(42.9)
*4–11 times in the last year*	102	(32.9)	105	(33.9)	119	(38.4)	115	(37.1)
*1–3 times in the last year*	30	(9.7)	31	(10)	30	(9.7)	25	(8.1)
*Never or not in the last year*	6	(1.9)	7	(2.3)	4	(1.3)	3	(1)
*Missing*	0	(0)	0	(0)	1	(0.3)	1	(0.3)
History of, *n* (%)								
*Heart Disease*	0	(0)	1	(0.3)	4	(1.3)	3	(1)
*High cholesterol*	26	(8.4)	20	(6.5)	25	(8.1)	12	(3.9)
*High blood pressure*	26	(8.4)	31	(10)	28	(9)	20	(6.5)
*Diabetes*	12	(3.9)	11	(3.5)	7	(2.3)	5	(1.6)
*Cancer*	5	(1.6)	9	(2.9)	6	(1.9)	7	(2.3)
*Chronic Obstructive Pulmonary Disease*	5	(1.6)	2	(0.6)	4	(1.3)	3	(1)

**Fig. 3:**
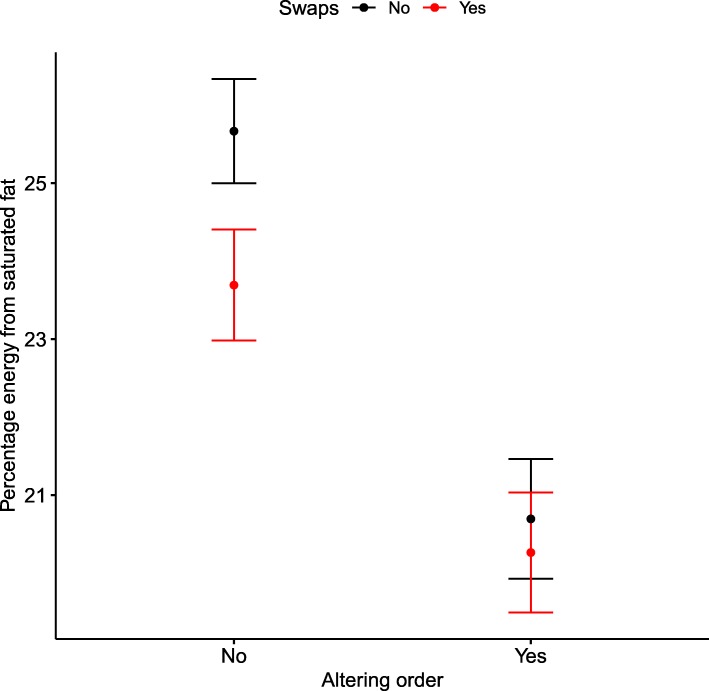
Mean (95% Confidence Intervals) percentage energy from saturated fat by group allocation (primary outcome)

On average, participants spent 21 min (SD 9) completing the study, browsed 29 pages of products (SD 15), and bought 12 products (SD 5). There were no significant differences in number of items or length of time to complete the task by group allocation (Additional file 2: Table S1). The percentage energy from SFA in the control shopping basket (*n* = 275) was 25.7% (±5.6%) (Fig. 3). Compared with no intervention, altering the default order of products to show foods in ascending order of SFA (*n* = 261) reduced SFA by − 5.0% (95%CI: − 6.3 to − 3.6)), and offering an explicit swap with lower SFA (*n* = 279) reduced it by − 2.0% (95%CI: − 3.3 to − 0.6). Altering the default order reduced the percentage energy from SFA significantly more than offering swaps (− 3.0% (95% CI: − 4.3 to − 1.6). The combined intervention (*n* = 273) was significantly more effective than control (− 5.4% (95%CI: − 6.7 to − 4.1)) and swaps alone (− 3.4% (95%CI: − 4.8 to − 2.1)), but there was no difference from altering the default order alone (− 0.4% (95%CI: − 1.8 to 0.9), *p* = 0.04 for interaction). These effects remained significant after a post-hoc analysis adjusting for multiple comparisons for the number of tests at an alpha of a = 0.05/28 = 0.001. The interaction between the two interventions was significant only for the primary outcome (p = 0.04). Interactions and main effects are presented in the Additional file 2: Table S2.

The proportion of products that contained less than 1.5% SFA in the shopping basket was significantly higher in each of the interventions compared with control. It was also significantly higher in the altering the default order group than for offering swaps (mean difference 6.5, 95% CI: 3.3 to 9.7) group. The cost of the shopping basket (£/100 g) did not significantly differ between groups (Table 2).

**Table 2:** Comparison of the secondary outcomes between trial groups

	Control	Swaps	Altering order	Combined	Swaps vs Control	Altering order vs Control	Combined vs Control	Altering order vs Swaps	Combined vs Swaps	Combined vs Altering order
	(*n* = 275)	(*n* = 279)	(*n* = 261)	(*n* = 273)						
% of products with < 1.5% SFA	20.4 ± 12.6	24.38 ± 13.45	30.87 ± 15.93	32.4 ± 16.15	3.98 (0.79 to 7.17)	10.47 (7.22 to 13.71)	12 (8.8 to 15.21)	6.48 (3.25 to 9.72)	8.02 (4.82 to 11.22)	1.54 (−1.71 to 4.79)
Cost, *£/100 g*	0.36 ± 0.12	0.37 ± 0.11	0.36 ± 0.1	0.36 ± 0.12	0.01 (− 0.01 to 0.04)	0 (−0.03 to 0.02)	0.01 (− 0.02 to 0.03)	− 0.01 (− 0.04 to 0.01)	−0.01 (− 0.03 to 0.02)	0.01 (− 0.02 to 0.03)
Total energy, *kcal*	14,467 ± 7038	14,393 ± 7923	12,448 ± 5894	12,670 ± 7574	−74 (− 1641 to 1493)	−2019 (− 3612 to − 425)	− 1797 (− 3372 to − 221)	− 1945 (− 3532 to − 357)	− 1723 (− 3292 to − 153)	222 (− 1374 to 1818)
Energy density, *kcal/100 g*	219.03 ± 47.52	211.12 ± 46.16	194.73 ± 45.96	190.76 ± 44.36	−7.91 (− 17.97 to 2.16)	−24.3 (− 34.53 to − 14.07)	−28.27 (− 38.38 to − 18.15)	−16.39 (− 26.59 to − 6.2)	− 20.36 (− 30.44 to − 10.28)	−3.97 (− 14.22 to 6.28)
Sugar, *% energy*	15.42 ± 4.99	15.39 ± 4.93	17.65 ± 6.48	17.82 ± 5.84	− 0.03 (− 1.25 to 1.19)	2.23 (0.99 to 3.47)	2.39 (1.17 to 3.62)	2.27 (1.03 to 3.5)	2.43 (1.21 to 3.65)	0.16 (− 1.08 to 1.41)
Salt, *g/100 g*	0.15 ± 0.13	0.15 ± 0.08	0.15 ± 0.07	0.14 ± 0.06	0 (− 0.02 to 0.02)	0 (− 0.02 to 0.02)	− 0.01 (− 0.03 to 0.01)	0 (− 0.02 to 0.02)	− 0.01 (− 0.03 to 0.01)	−0.01 (− 0.03 to 0.01)

Values are means ± SDs in the first four columns and mean differences (95% CIs) in the next six columns. *SFA* Saturated fat

Altering the default order of products alone or in combination with swaps significantly reduced the total energy and energy density of the shopping basket compared with no intervention or swaps alone. Altering the order and the combination of the interventions significantly increased the percentage energy from total sugars compared to swaps alone or control, whereas swaps alone did not significantly change the percentage energy from total sugars compared to control. There was no evidence of a difference in salt between any of the four study groups (Table 2).

**Fig. 4:**
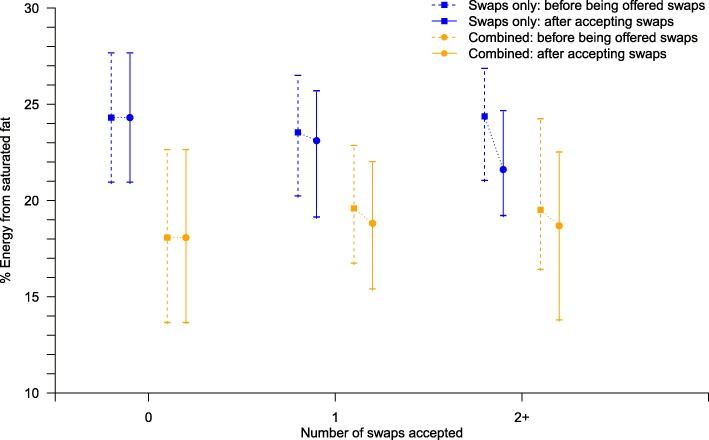
Median (interquartile range) of percentage energy from saturated fat of the basket before being offered swaps and after accepting swaps by number of swaps accepted

The percentage of participants who were offered at least one swap was 100 and 94% in the swap alone group and combined group, respectively. The swaps alone group was offered a median of 7 (IQR 4) swaps whereas the combined group was offered 4 (IQR 4) swaps. The percentage of participants who accepted at least one swap was 63 and 40% in the in the swap alone group and combined group, respectively (Additional file 2: Table S3). The change in percentage energy from SFA (the primary outcome) increased with the number of swaps accepted as shown in Fig. 4. These results did not differ significantly by the pre-specified subgroups of sex, age, ethnic group, BMI, education, or income (Table 3 and Additional file 2: Table S4).

**Table 3:** Subgroup analyses of the percentage of energy from saturated fat (primary outcome) by trial group

	% energy from saturated fat (mean ± SD, n)				Between group mean difference (95% CI)		
	Control	Swaps	Altering order	Combined	Altering order vs Swaps	Combined vs Swaps	Combined vs Altering order
Sex							
*Female*	25.58 ± 5.62, 172	24.3 ± 6.08, 183	21.26 ± 6.12, 179	21.11 ± 6.09, 174	− 3.04 (− 4.66 to − 1.42)	−3.19 (− 4.82 to − 1.55)	− 0.14 (− 1.78 to 1.5)
*Male*	25.81 ± 5.69, 103	22.54 ± 5.81, 96	19.59 ± 6.55, 80	18.72 ± 6.91, 96	− 2.95 (− 5.39 to − 0.51)	− 3.82 (− 6.14 to − 1.5)	− 0.87 (− 3.31 to 1.57)
Age, *years*							
*Median and above*	25.74 ± 5.89, 142	22.9 ± 6.56, 144	21.54 ± 6.35, 134	21.03 ± 6.51, 131	− 1.36 (− 3.32 to 0.59)	−1.87 (− 3.84 to 0.1)	− 0.51 (− 2.51 to 1.5)
*Below median*	25.59 ± 5.37, 133	24.54 ± 5.32, 135	19.81 ± 6.15, 127	19.56 ± 6.34, 142	−4.73 (− 6.58 to − 2.88)	−4.98 (− 6.78 to − 3.18)	−0.25 (− 2.08 to 1.58)
Ethnic group							
*White*	25.66 ± 5.73, 240	23.77 ± 5.88, 256	20.8 ± 6.29, 241	20.22 ± 6.43, 244	− 2.96 (− 4.37 to − 1.56)	−3.55 (− 4.95 to − 2.15)	−0.59 (− 2.01 to 0.84)
*Non-White*	25.74 ± 4.97, 35	22.94 ± 7.85, 22	19.44 ± 6.57, 19	20.65 ± 6.81, 28	−3.5 (− 8.78 to 1.78)	−2.29 (− 7.09 to 2.51)	1.21 (− 3.8 to 6.22)
BMI, *kg/m*^*2*^							
*< 30*	25.86 ± 5.31, 199	23.72 ± 6.14, 195	20.11 ± 6.35, 170	20.64 ± 6.51, 200	−3.61 (− 5.26 to − 1.97)	−3.08 (− 4.66 to − 1.5)	0.53 (− 1.11 to 2.17)
*≥ 30*	25.44 ± 6.14, 73	23.61 ± 5.86, 83	22.12 ± 6.04, 86	19.39 ± 6.14, 69	−1.5 (− 3.9 to 0.9)	− 4.22 (− 6.76 to − 1.68)	− 2.72 (− 5.24 to − 0.2)
Education							
*None/Secondary*	25.09 ± 5.83, 68	23.52 ± 6.49, 103	20.07 ± 5.87, 99	19.89 ± 6.69, 79	−3.46 (− 5.73 to − 1.19)	−3.63 (− 6.04 to − 1.22)	−0.17 (− 2.6 to 2.26)
*Higher*	25.86 ± 5.57, 207	23.79 ± 5.79, 175	21.1 ± 6.55, 161	20.41 ± 6.38, 193	−2.7 (− 4.4 to − 0.99)	−3.38 (− 5.01 to − 1.75)	− 0.69 (− 2.35 to 0.98)
Household income							
*Low-middle (£25-39 k)*	25.42 ± 5.57, 191	23.21 ± 6.28, 188	20.36 ± 6.29, 181	19.81 ± 6.3, 177	− 2.85 (− 4.49 to − 1.21)	− 3.4 (− 5.05 to − 1.76)	− 0.55 (− 2.22 to 1.11)
*Higher (£40 k+)*	26.2 ± 5.79, 75	24.86 ± 5.38, 84	21.67 ± 6.25, 70	21.6 ± 6.54, 86	− 3.2 (− 5.71 to − 0.69)	−3.26 (− 5.64 to − 0.88)	−0.07 (− 2.56 to 2.43)

The median acceptance rate was 14 and 0% in the swaps alone and combined intervention, respectively (Table 4). There were no between-group differences in the acceptance rate by specific food groups, i.e. butter, cheese, milk, meat, and sweets. The median proportion of accepted swaps out of the total bought items was not different between the swaps alone and combined interventions.

**Table 4:** Comparison of outcomes applicable only between swaps alone and combined groups

	Swaps		Combined		Median difference (95% CI)
	Median (IQR)	*n*	Median (IQR)	*n*	
% swaps accepted out of swaps offered					
*total*	14.3 (0–28.57)	279	0.0 (0–25)	273	14.29 (0 to 2.78)
*High SFA change*^a^	23.5 (14.3–37.5)	167	33.3 (20–50)	99	− 9.80 (− 13.33 to − 4.17)
*Low SFA change*^a^	0.0 (0–0)	112	0.0 (0–0)	174	0.00 (0 to 0)
*Cheese*	0.0 (0–100)	279	0.0 (0–0)	273	0.00 (0 to 0)
*Butter, margarine, spreads*	0.0 (0–50)	279	0.0 (0–0)	273	0.00 (0 to 0)
*Sweets and desserts*	0.0 (0–33.33)	279	0.0 (0–20)	273	0.00 (0 to 0)
*Milk*	0.0 (0–0)	279	0.0 (0–0)	273	0.00 (0 to 0)
*Meat*	0.0 (0–0)	279	0.0 (0–0)	273	0.00 (0 to 0)
% of accepted swaps out of total number of basket items	20.0 (15.4–27.27)	105	18.2 (14.9–22.44)	32	1.82 (− 1.39 to 4.78)

*SFA* Saturated fat. ^a^proportion of swaps accepted out of those offered by median observed change in SFA (high SFA change vs low SFA change). The majority of participants were offered a maximum of one swap per product category, as they were intrusted to buy only one product. Therefore, the percentage of swaps accepted out of those offered would have a value of either 0% or 100% in most cases. There was more variation in desserts, as participants bought more than one desserts

Following completion of the task, participants reported that the three most important factors to be considered when choosing foods or drinks to buy were price, taste, and healthiness (Additional file 2: Table S5). These were also reflected in the open-ended comments. About a quarter (24%) reported checking the nutrition labels for saturated fat content either often or always (Additional file 2: Table S6). About three quarters (76%) of those in the groups that were offered swaps agreed that swaps are a feature that they would like to see in their usual shopping with only 10% of them disagreeing and the rest being indifferent to this feature. About 17% (*n* = 187) left open-ended comments. Although we did not ask about the acceptability of altering the default order, both interventions emerged as mostly acceptable in the comments. (“*The auto healthy swap option is brilliant as many people would rather have the healthier option given the choice and often don’t have the time or patience to compare products.*”, “*When looking at food categories, the top few results seemed to always be the lighter/healthier options - this is a good idea!*”). A minority of participants (*n* = 8) commented that the shopping list did not reflect their habitual purchasing pattern, because they usually buy less treats or cook from scratch (“*The shopping list was very unrealistic for me. We mostly cook from scratch and rarely buy ready meals or desserts”).* Further comments evolved around the functionality of the website (“*The site was slow when I was choosing the product but everything went well*”), the enjoyment of taking part in the study (“*Very interesting study, I enjoyed taking part”*), and factors influencing their usual shopping and eating behaviours, which resembled the factors captured with the quantitative data (“*As a household, we food shop to keep the costs low*”).

## Discussion

Altering the default order to show foods in ascending order of SFA and offering an explicit swap with lower SFA during an online shopping experience significantly reduced the percentage energy from SFA of the selected foods. Altering the default order was significantly more effective than offering swaps and there was no evidence that providing swaps in addition to altering the order augmented the effect of the intervention. There was no evidence that the intervention affected the expected cost of products selected.

This is the first randomised trial aiming to directly compare an environmental-level (altering the default order) and an individual-level (offering swaps) intervention and their combination for improving the nutrition quality of food purchases. A previous trial conducted in a real online supermarket testing swaps with lower SFA demonstrated a much smaller effect than observed in the current trial [9]. However, their primary outcome was calculated as grams of SFA per total weight of foods in the total basket rather than as a percentage of energy. Whether any difference in effect size is due to the simulated versus real environments or due to differences in the shopping lists, the algorithm suggesting the swap, or the number of swaps accepted is unclear. Our interventions to reduce percentage energy from SFA also reduced the energy density of items selected. This is in contrast with a previous study using the same experimental platform which specifically offered swaps with lower energy density but found no effect of the intervention. This might be explained by the differences in the shopping lists, in the algorithm determining swaps, or the exclusion of energy dense cooking ingredients, such as butter, from their analysis [12]. A previous systematic review of interventions to change food purchasing behaviours in grocery stores did not identify any other randomised controlled trials on the effect of altering the default order of foods [22]. However, a recent trial showed no difference of altering the positioning of a specific category of foods (i.e. fruit and vegetable snacks) on the proportion of orders in an online school canteen ordering system. The discrepancy with our findings might be because that intervention was trying to make more prominent a category of food which people were not necessarily trying to buy, whereas we were trying to make certain foods more prominent within a specific category from which participants had been instructed to select an item [23].

There was a small increase in the proportion of sugars in the altering order and combined groups, which reflects differences in the formulation of products rather than a specific effect of the intervention. In this experiment, the effect was mostly attributable to the composition of the dessert items where the items containing less SFA tended to have a higher proportion of sugars. Furthermore, altering order and combined groups also resulted in a large reduction of in total energy (~ 2000 kcal on average). This effect may outweigh the small increase in the proportion of total sugars and suggests the potential additional contribution of these interventions to cardiovascular risk reduction through reductions in energy intake. There was no difference in salt between groups, but the average salt percentage in the basket was well below the cut-off of 0.3% that qualifies a food product as a low in salt (‘green’ in the traffic light system).

Individual-level interventions, such as providing healthier swaps, may require more cognitive resource than environmental-level interventions targeting automatic processes [24]. Under the theory of the dual-system model, our environmental-level intervention (altering the order) fits under the “System 1” whereby processes are automatic and intuitive. In contrast, the individual-level intervention (offering swaps) fits under the “System 2” whereby processes are slow, rule-based, and analytical [24]. Our study shows that an intervention targeting the effortless System 1 processes leads to larger effect sizes, irrespective of the presence of interventions targeting the effortful System 2 processes. However, the System 2 manipulation happened after the initial choice which may have limited its potential effectiveness compared to the System 1 manipulation that occurred before the initial choice was made. Alternatively, this outcome may be explained by the smaller number of swaps offered in the combined intervention compared with the swaps alone. Furthermore, it has been suggested that environmental-level interventions to promote healthier eating may be more effective in reducing health inequalities, in contrast with individual-level ones that require more individual agency and greater cognitive resource [25]. To the best of our knowledge, this is the first study to compare interventions of both systems separately and in combination to facilitate healthy eating [26].

There has been a sharp rise in the proportion of households purchasing groceries online, reaching 28% of UK consumers in 2017 from 4% a decade earlier, with similar upward trajectories being observed across Europe [27], East Asia [28], and the USA [29]. This trial shows that offering swaps and altering the default order are potentially effective strategies to encourage healthier food purchasing. Real online supermarkets usually alter the position of foods for marketing purposes, but to our knowledge not for health purposes. A UK online supermarket is already implementing healthier swaps at checkout, but the algorithm is unpublished and a previous trial suggested higher acceptance of swaps at the point of selection than at checkout [12]. In our study, there was some indication that swaps for cheese, butter, and sweets and desserts might have been more acceptable than those for milk or meat. Future research could examine whether swaps are more acceptable in certain product categories.

Future research should also aim to test these strategies using real online platforms and supermarkets and, contingent on their effect in the short term, study their long-term impact on food purchasing habits. In the meantime, online supermarkets should be encouraged to play a more proactive role in shaping heathier choices for their customers and can capitalise on the results of this study by offering either or both interventions knowing that they are potentially effective strategies for reducing percentage energy from SFA without increasing cost to their customers.

Strengths of this study include the randomised factorial design, blinded statistical analysis, high completion rate, precision of the estimate of the treatment effect, and the use of products typically present in real supermarkets in a convincing simulated grocery store website. Open text responses from a small number of participants at the end of the study implied that participants had fully engaged with the shopping experience.

Limitations of the study include the use of an experimental research platform in which participants neither spent their own money nor received the food they chosen. The shopping list in the trial comprised food categories of products, that although typically bought, were focused on foods which are sources of SFA. Therefore, the effect of the swap intervention is likely to be smaller during a real shopping experience where a broader range of products are likely to be purchased. The experimental nature of the study might have overestimated the effect of altering the order of products, as the desire to perform the experiment rapidly without much thought to foods being selected might have led to a bias towards choosing the items at the top of the list. However, our process evaluation showed that participants spent about 14–18 min on average in the supermarket to buy 11 products and this time period was similar across all groups. Participants also browsed about three times more pages than the number of products they added to the basket in each condition. This potential bias might be quantified in future trial through the use of eye-tracking technology [30]. Bias towards the lower saturated fat option to meet the study aims (demand characteristics) is plausible, especially in the swap interventions. However, the acceptance rate of swaps was relatively low (14% in the swap group and 0% in the combined) to suggest this risk is low. Participants who did not buy exactly 10 items as instructed were included in the analysis, but the proportion of these participants did not differ by group. All the above process evaluation measures highlight the validity of the supermarket task. We sent invitations for study participation to a random subsample from a pool of 6968 participants who had all been pre-screened as eligible, but we were not able to record the invitation and response rates to the study, which may limit the generalisability of our findings. BMI was similar to national average. However, the recruitment pool and, subsequently, study participants reported higher than average education and participants reported spending more money on weekly food shopping (£70) than the national average (median: £58) [31]. Although we did not observe differences in the effectiveness of the intervention based on socio-economic status or demographic characteristics, our ability to detect differences may be limited by the characteristics of the sample and because we did not power the study to detect subgroup differences.

## Conclusions

In conclusion, altering the default order to show foods in ascending order of SFA and offering a swap with lower SFA reduced percentage energy from SFA in an experimental online supermarket. Environmental-level interventions, such as altering the default order, may be a more promising way to improve food purchasing than individual-level ones, such as offering swaps.

## Additional files

Additional file 1: Study protocol and statistical analysis plan. (PDF 716 kb)

Additional file 2: Supplementary figures and tables (Description of data: Examples of the interventions and additional analysis). (PDF 78 kb)

## Data Availability

The datasets used and/or analysed during the current study are available from the corresponding author on reasonable request.

## Abbreviations

REDCap: Research Electronic Data Capture
SFA: Saturated fat
