# Supplementary material for: Prominent positioning and food swaps are effective interventions to reduce the saturated fat content of the shopping basket in an experimental online supermarket: a randomized controlled trial
Source: Int J Behav Nutr Phys Act. 2019 Jun 7;16:50. doi: 10.1186/s12966-019-0810-9 (PMC6555993; doi:10.1186/s12966-019-0810-9)
Supplement: Supplementary file 1 — Study protocol and statistical analysis plan. (PDF 716 kb) [file 12966_2019_810_MOESM1_ESM.pdf]

Additional file 1 – Study protocol (Page 2) and statistical analysis plan (Page 28)

Study Title: The effectiveness of individual-level and environmental-level interventions on food choices: an experimental online supermarket study

Internal Reference Number / Short title: The OLS Study

Med IDREC Ref: R55722/RE001

Date and Version No: 13-Jul-2018, version 3.0

Chief Investigator: Dr Carmen Piernas-Sanchez[1]

carmen.piernas-sanchez@phc.ox.ac.uk, 01865 289284

Nuffield Department of Primary Care Health Sciences, University of Oxford

Investigators: Professor Susan Jebb[1]

Professor Paul Aveyard[1]

Dr Dimitrios Koutoukidis[1]

Mrs Sarah Payne-Riches[1]

Dr. Jose Ordoñez-Mena[1]

[1]Nuffield Department of Primary Care Health Sciences, University of Oxford

Sponsor: University of Oxford

Funder: NIHR Biomedical Research Centre (BRC) and Collaboration for Leadership in Applied Health Research and Care (CLARHC)

Chief Investigator Signature:

Conflicts of interest

None declared.

Confidentiality Statement

This document contains confidential information that must not be disclosed to anyone other than the authorised individuals from the University of Oxford, the Investigator Team and members of the Medical Sciences Interdisciplinary Research Ethics Committee (Medical Sciences IDREC), unless authorised to do so.

TABLE OF CONTENTS

1. SYNOPSIS 4
2. ABBREVIATIONS 6
3. BACKGROUND AND RATIONALE 7
4. OBJECTIVES AND OUTCOME MEASURES 9
5. STUDY DESIGN 11
6. PARTICIPANT IDENTIFICATION AND RECRUITMENT 12
   6.1. Study Participants 12
   6.2. Inclusion Criteria 12
   6.3. Exclusion Criteria 12
7. INTERVENTIONS 13
8. STUDY PROCEDURES 15
   8.1. Recruitment 15
   8.2. Screening and Eligibility Assessment 15
   8.3. Informed Consent 15
   8.4. Randomisation 15
   8.5. Blinding 15
   8.6. Baseline Assessments 16
   8.7. Follow-up 16
   8.8. Discontinuation/Withdrawal of Participants from Study 16
   8.9. Definition of End of Study 16
9. STATISTICS AND ANALYSIS 16
   9.1. The Number of Participants 16
   9.2. Analysis of Outcome Measures 16
10. DATA MANAGEMENT 17
   10.1. Access to Data 17
   10.2. Data Handling and Record Keeping 17
11. QUALITY CONTROL AND QUALITY ASSURANCE PROCEDURES 17
12. ETHICAL AND REGULATORY CONSIDERATIONS 17
   12.1. Declaration of Helsinki 17
   12.2. Approvals 17
   12.3. Participant Confidentiality 17
   12.4. Expenses and Benefits 18

12.5. Annual Progress Report 18
12.6. Other Ethical Considerations 18
13. FINANCE AND INSURANCE 18
13.1. Funding 18
13.2. Insurance 19
14. PUBLICATION POLICY 19
15. REFERENCES 20
16. APPENDIX A: STUDY FLOW CHART 21
17. APPENDIX B: SCHEDULE OF STUDY PROCEDURES 22
18. APPENDIX C: AMENDMENT HISTORY 27

1. SYNOPSIS

**Study Title**	The effectiveness of individual-level and environmental-level interventions on food choices: an experimental online supermarket study	
**Internal ref. no.**	N/A	
**Study Design**	Randomised controlled trial with a 2x2 factorial design	
**Study Participants**	Volunteers from the UK	
**Planned Sample Size**	1240	
**Planned Study Period**	February 2018 – January 2019	
	**Objectives**	**Outcome Measures**
**Primary**	To investigate the magnitude of saturated fat achieved in the shopping basket in response to an individual-level intervention and an environmental-level intervention, separately and in combination, compared to control (no intervention).	Difference in the saturated fat of the final basket (measured in % of total energy) between each of the four trial arms
**Secondary**	To investigate the effect of the interventions on:<br>i. the proportion of products with less than 1.5g saturated fat/100g in the final basket<br>ii. the overall cost of the final shopping basket<br>iii. the diet composition of the shopping basket<br><br>To investigate the effect of the individual-level intervention on<br>iv. saturated fat change per swap accepted<br>v. the proportion of swaps accepted out of those offered overall<br>vi. the proportion of swaps accepted out of those offered by magnitude of reduction<br>vii. the proportion of swaps accepted out of those offered by type of food group<br>viii. the proportion of accepted swaps out of total shopping basket items	Between each trial arm:<br>i. difference in the proportion of products with lower saturated fat in the final basket (%)<br>ii. difference in the overall cost of the final shopping basket (£) weighted for the size of the basket (g)<br>iii. difference in the total energy, energy density, sugars (% energy) and salt (g/100g) content of the shopping basket<br><br>Between the single individual-level intervention (swaps only) and the combined intervention arms<br>iv. difference in % saturated fat content per swap accepted (% energy intake)<br>v. difference in the proportion of swaps accepted out of those offered (%)<br>vi. difference in the proportion of swaps accepted out of those offered by median observed change in saturated fat

		vii. difference in % swaps accepted out of those offered (%) for (a) butter, margarine, and spreads, (b) cheese, (c) milk, (d) meat, and (e) sweets and desserts<br>viii. difference in the proportion of accepted swaps out of total shopping basket items (%)

2. ABBREVIATIONS

BRC	Biomedical Research Centre
CI	Chief Investigator
CLARHC	Collaboration for Leadership in Applied Health Research and Care
CVD	Cardiovascular Disease
CTRG	Clinical Trials & Research Governance, University of Oxford
IDREC	Interdisciplinary Research Ethics Committee
LDL	Low-Density Lipoprotein
SFA	Saturated Fat

3. BACKGROUND AND RATIONALE

Poor diet is a major contributor to cardiovascular disease (CVD). Saturated fat (SFA) increases the production of low-density lipoprotein (LDL) cholesterol and decreases its clearance from the body through suppression of the LDL receptor activity [1]. A recent Cochrane systematic review and meta-analysis showed that interventions to reduce SFA intake compared to usual diets resulted in a significant reduction in LDL cholesterol of 0.19 mmol/L [-0.33,-0.05] and 17% reduction in CVD risk [2]. However, while the evidence is clear about what should be done, it is far less clear that we have practical means of achieving these dietary changes at the population level. Progress in reducing SFA through public education programmes is slow, and SFA intake in the UK (13.5% energy) remains more than a third higher than the recommended value of <10% energy.

Previous research has established that it is possible to achieve lower SFA intake through providing appropriate food stuffs in place of higher SFA products, in combination with intensive and tailored counselling [3, 4]. However, the success of dietary advice alone to reduce SFA has been limited and has only been achieved with specialist staff and high intensity behavioural support [5, 6]. It is increasingly recognised that the health system needs a mechanism to support a large number of people classified as at increased CVD risk to change their diet, but as yet there is no effective intervention that is sufficiently scalable and practical for routine delivery in routine care settings. There is very limited evidence suggesting that brief dietary counselling in a primary care setting can be beneficial on health and specifically on blood cholesterol [7]. However, the effects are modest and, as with other interventions requiring high levels of agency, may be more effective in some population groups than others [8]. An approach that also includes environmental restructuring may reduce the risk of widening inequalities and may be more successful in sustaining individual behaviour change.

Food purchasing is a key antecedent of food consumption, therefore individual-level interventions targeting the nutritional quality of the grocery shopping present a clear opportunity for action. Effective intervention approaches for individual dietary change identified in systematic reviews include providing tailored dietary advice, information, self-monitoring and personalized feedback [9, 10]. The use of new technologies can easily incorporate these and other elements to deliver a personalised behaviour change intervention, especially in the context of an online supermarket. For example, recommending lower SFA options at the point of purchase based specifically on items added to the basket has shown a significant reduction in total SFA from online food purchases with no difference in price paid [11].

There is growing interest in structural interventions in everyday contexts to change eating behaviours by altering the defaults at the point of choice, so called choice architecture interventions [12]. This recognises that, in practice, many decisions about food are not reflective, conscious choices but are automatic reactions prompted by environmental cues [13]. Evidence suggests that these “nudging” interventions might achieve a meaningful impact on behaviours and could be applied in the retail environment to influence food purchasing [9]. We hypothesise that the impact of environmental interventions can be enhanced by individual-level interventions, especially among those motivated to change.

Online supermarkets offer unique opportunities to deliver and support complex nutrition interventions to shape food purchasing patterns at scale, but research in this arena is still in its infancy and more solid evidence is required to develop a truly effective intervention with population-level impact. The proposed

research is especially timely; while online grocery shopping accounts for 10-30% in most developed countries [14], it is rapidly expanding. Currently, more than a fifth of British households from a broad socio-economic spectrum are buying online groceries every month and this figure is expected to double over the next five years [15].

The purpose of this project is to test the effects of an individual-level intervention and an environmental-level intervention, separately and in combination, on SFA in a representative sample of UK adults. If effective, similar interventions could be offered by supermarkets to all their customers to bring health benefits to the whole population and encourage supermarkets to play a more proactive role in shaping healthier choices for their customers. We do not anticipate unintended or adverse effects due to the intervention. These proposed interventions are low cost and could reach large numbers, meaning that they could have a significant population impact and be very cost-effective, even if the effect size is smaller than more intensive interventions.

4. OBJECTIVES AND OUTCOME MEASURES

Objectives	Outcome Measures
**Primary Objective**<br>To investigate the magnitude of saturated fat achieved in the shopping basket in response to an individual-level intervention and an environmental-level intervention, separately and in combination, compared to control (no intervention).	Difference in the saturated fat of the final basket (measured in % of total energy) between each of the four trial arms
**Secondary Objectives**<br>To investigate the effect of the interventions on:<br>i. the proportion of products with less than 1.5g saturated fat/100g in the final basket<br>ii. the overall cost of the final shopping basket<br>iii. the diet composition of the shopping basket<br><br>To investigate the effect of the individual-level intervention on<br>iv. saturated fat change per swap accepted<br>v. the proportion of swaps accepted out of those offered overall<br>vi. the proportion of swaps accepted out of those offered by magnitude of reduction<br>vii. the proportion of swaps accepted out of those offered by type of food group<br>viii. the proportion of accepted swaps out of total shopping basket items	Between each trial arm:<br>i. difference in the proportion of products with lower saturated fat in the final basket (%)<br>ii. difference in the overall cost of the final shopping basket (£) weighted for the size of the basket (g)<br>iii. difference in the total energy, energy density, sugars (% energy) and salt (g/100g) content of the shopping basket<br><br>Between the single individual-level intervention (swaps only) and the combined intervention arms<br>iv. difference in % saturated fat content per swap accepted (% energy intake)<br>v. difference in the proportion of swaps accepted out of those offered (%)<br>vi. difference in the proportion of swaps accepted out of those offered by median observed change in saturated fat<br>vii. difference in % swaps accepted out of those offered (%) for (a) butter, margarine, and spreads, (b) cheese, (c) milk, (d) meat, and (e) sweets and desserts

	viii. difference in the proportion of accepted swaps out of total shopping basket items (%)
**Non-efficacy**<br>To investigate the acceptability of each intervention.	Rating scores and open-ended answers from the follow-up questionnaire

5. STUDY DESIGN

This is a 2x2 factorial randomised controlled trial of an individual-level and an environmental-level intervention to reduce the SFA content of the total shopping basket during an online shopping experiment. Participants will be recruited through research agencies. Each participant will be randomised to one of the four trial arms and participate online for about 30 minutes. We will collect and analyse data from the online platform used during the intervention and from follow-up feedback questionnaires (see Appendix A for study flow chart).

6. PARTICIPANT IDENTIFICATION AND RECRUITMENT

6.1. Study Participants

Eligible participants will be UK adultswho are the main (or shared) grocery shopper for their household, able to read and understand the instructions provided and to provide consent to take part in the study. In addition, participants have to be and have access to a computer and the Internet.

People who have any dietary restriction will not be eligible to participate.

6.2. Inclusion Criteria

- UK adults, aged ≥18 years.
- Able to speak and read English.
- Willing and able to give informed consent for participation in the study.
- Being the main (or shared) grocery shopper for their household.
- Having access to a computer and Internet.

6.3. Exclusion Criteria

The participant may not enter the study if ANY of the following apply:

- Having any dietary restriction.

7. INTERVENTIONS

This study uses a bespoke virtual online supermarket shopping (OLS) platform, hosted by The University of Oxford, which emulates a real online supermarket for research purposes relating to food purchasing interventions (www.woodssupermarket.co.uk). It contains a food database downloaded from a real UK grocery retailer (Tesco.com API. February 2012) with ~11,000 products. Nutrient information was supplemented by manual linkages with food labels with data provided by Kantar WorldPanel and the MRC Human Nutrition Research food and nutrient database.

The shopping task will involve shopping to a pre-determined shopping list of approximately 10 items. Initial testing of the platform showed that the reliability was greater with a list-based task compared to free choice, hence a food list will be provided to the participants for the shopping task [16]. The food items included in the list have been chosen so that they offer opportunities to purchase items that are the major food sources of SFA in the UK: dairy (e.g. milk/cheese), meat (e.g. beef) and cereal products (e.g. biscuits); but are also within food categories where lower SFA options are available to choose from.

Food shopping list for the participants in the study:

- Milk for everyday use
- Butter or margarine for everyday use
- Cheese for use in a sandwich or light meal
- Ready-to-eat savoury entree item
- Ready-to-eat individual chilled desserts
- Meat/fish/vegetarian alternative to cook for 4 people
- Dessert for a meal for 4 people
- Something to eat with a hot drink
- A sweet snack item to eat now
- A savoury snack item to eat now

Participants will be asked to click the link through to the supermarket shopping platform to begin the task. The shopping task is estimated to take participants 30 minutes to complete. Participants will only be asked to complete the shopping task once.

Participants will be provided with instructions as follows:

"*We would like you to do some online grocery shopping on a supermarket website. This is not a real supermarket, and you will not be asked to spend your own money.*

We will give you a shopping list and ask you to buy all the items on the list. You do not need to buy additional items to serve with these foods or items from your usual shopping list.

When doing your shopping, try to imagine you are doing your own grocery shopping and choose foods that you and your household would eat. You should choose the things you normally buy or wouldn't mind eating."

Participants will be randomly allocated to one of the following groups when shopping online:

1) Individual-level intervention: Offering a swap to a product with less SFA

Swaps will be offered at point of selection i.e. when a participant selects an item to put in their shopping basket, if an alternative product exists that is lower in SFA within the same food category, the participant will be offered the chance to swap the item. Products offered as swaps will be within the same general price and weight range as the original item.

2) Environmental-level intervention: Prominent positioning of lower SFA options
This will apply to each list of foods offered to participants when searching for products.
3) A combination of individual- and environmental-level interventions
This group will receive both intervention as described above.
4) Control
Participants in this group will see the default version of the website with no swaps offered and a random order of the foods displayed in response to each search.

8. STUDY PROCEDURES

8.1. Recruitment

National research agency panels [either ResearchNow (https://www.researchnow.com/), Prolific (https://www.prolific.ac/), or both] will screen and recruit a sample of volunteers.

8.2. Screening and Eligibility Assessment

Panel members will be sent an email introducing the study and including a link to the participant information sheet. Panel members who are interested in taking part in the study will be asked to click on a web-link from the email, which will take them to the study registration website. At this registration website, they will be asked screening questions indicating their responsibility for shopping, if they have any dietary restriction, and to confirm their age and country of residence. They will also be able to re-read the detailed participant information before consenting to participate.

8.3. Informed Consent

After reading the detailed participant information sheet and answering screening questions to confirm they meet the inclusion/exclusion criteria; and if they are happy to proceed, participants will be asked to give consent electronically to take part in the study.

Written electronic versions of the Participant Information and Informed Consent will be presented to the participants detailing no less than: the exact nature of the study, what it will involve for the participant and the implications and constraints of the protocol. It will be clearly stated that the participant is free to withdraw from the study at any time for any reason and with no obligation to give the reason for withdrawal.

The participant will be allowed as much time as wished to consider the information, and the opportunity to question the Investigator, or to discuss with others before deciding whether they wish to participate in the study. Electronic Informed Consent will then be obtained by means of clicking on a tick box.

8.4. Randomisation

Randomisation will be performed by the survey platform via computerised random number generation on an 1:1:1:1 basis with random block sizes and participants will be directed to a website that introduces an online shopping task. Allocation concealment is achieved, as participants are recruited from independent research panels and are being directed for automatic randomisation in the survey platform.

8.5. Blinding

Investigators will be not be blinded to intervention allocation but they will not be able to manipulate any study parameter following the initial study set up, as all study procedures are taking part in the online platform. The outcome assessment is blinded, as it happens automatically in the online platform. The statistician who will analyse the data will be blinded to intervention allocation. Due to the nature of the intervention, it will not be possible to blind participants to the intervention. However, participants will only be aware of the trial arm that they are exposed to and will be unaware of the other trial arms.

8.6. Baseline Assessments

Following consent, participants will complete a baseline questionnaire on demographic, shopping, and health data (Appendix B).

8.7. Follow-up

Participants will complete a short post-intervention survey (Appendix B) about the acceptability of the two interventions in the online shopping task and their usual shopping behaviours.

8.8. Discontinuation/Withdrawal of Participants from Study

Each participant has the right to withdraw from the study at any time. Participants will not be replaced as we will recruit sufficient sample size to allow for non-completion rate. The reason for withdrawal will be recorded. Withdrawal from the study, based on the pre-defined completion criteria (section 9.2), will result in exclusion of the data for that participant from analysis.

8.9. Definition of End of Study

The end of study is the completion of the shopping task and survey of the last participant.

9. STATISTICS AND ANALYSIS

9.1. The Number of Participants

There are no previously reported standard deviations of the mean difference from similar trials to guide the estimation of the standard deviation, and, thus, the calculation of the sample size.

Therefore, we conducted an initial pilot to allow for the power calculation. The initial plan was to recruit 500 people for the pilot and include those in the analysis of the full trial. Due to logistical constraints, 129 people were recruited for an independent pilot. This sample included 31-33 participants per arm with valid data (i.e. people who bought at least 5 items from the list) and showed a standard deviation of 6.5%.

If we were to observe a minimally clinically relevant 2% reduction in SFA (assuming 7% standard deviation) in the total basket between any of the 4 groups using intention to treat analyses with 90% power and two-sided $\alpha=0.05$, we would require 258 per group giving a total of 1032 participants. A final sample of 1240 participants would account for 20% attrition through participants not completing the shopping task.

9.2. Analysis of Outcome Measures

Two-way ANOVA will be used to test for

- SFA in each intervention group compared to control;
- SFA in the combined intervention group compared to single intervention groups
- SFA in one single intervention group versus the other single intervention group

- SFA in the combined intervention group compared to control.

Completion of the task will be defined as purchase of at least 5 out of 10 items listed in section 7.

Please refer to the Statistical Analysis Plan (v1.0, 13-Jul-2018) for details on the analysis. The SAP has been finalised before conducting the statistical analysis, therefore all analyses has been pre-defined.

10. DATA MANAGEMENT

10.1. Access to Data

Direct access will be granted to authorised representatives from the University of Oxford for monitoring and/or audit of the study to ensure compliance with regulations.

10.2. Data Handling and Record Keeping

All study data will be captured in a password protected secure database. The participants will be identified by a unique study specific number and/or code in any database. Personal data will not be collected in this study. Research data and records will be retained for at least three years after publication or public release of the work of the research and reviewed thereafter.

11. QUALITY CONTROL AND QUALITY ASSURANCE PROCEDURES

The study will be conducted in accordance with the current approved protocol, relevant regulations and standard operating procedures.

12. ETHICAL AND REGULATORY CONSIDERATIONS

12.1. Declaration of Helsinki

The Investigator will ensure that this study is conducted in accordance with the principles of the Declaration of Helsinki.

12.2. Approvals

The protocol, informed consent form, participant information sheet and any proposed advertising material will be submitted to the ethical committee for approval.

The Investigator will submit and, where necessary, obtain approval from the above parties for all substantial amendments to the original approved documents.

12.3. Participant Confidentiality

All data collected by participants will be anonymous. The participants will be identified only a participant ID number on all study documents and any electronic database. All documents will be stored securely requiring password-protected access and will be only accessible by study staff and authorised personnel.

Privacy and confidentiality of data is particularly hard to manage in Internet-based research because as researchers we are not in control of online communication networks, leading to the risk of third-party interceptions. We will encourage the use of a secure link at the consent stage. Participants will be informed that we will not collect their IP addresses. As the data will be anonymised, it does not constitute personal data and the duties and obligations of the Data Protection Act do not apply. The research agencies will only share with the researchers a participant ID and no personal data.

12.4. Expenses and Benefits

Participants recruited within these panels are rewarded through a different schemes. At the ReserachNow panel, panellists are rewarded for taking part in the task according to a structured incentive scheme, with the incentive amount offered for a survey determined by the length of survey and nature of the sample. All incentives are awarded only once the survey has been completed. The incentive options allow panellists to redeem from a large range of gift cards, points programs, charitable contributions, and partner products or services. At the Prolific panel, participants will be rewarded with £5 for qualifying for and completing the survey.

12.5. Annual Progress Report

The CI shall submit on request, a Progress Report to the Medical IDREC with a copy to CTRG.

12.6. Other Ethical Considerations

Internet based research issues

a) Authentication

The research agencies who will recruit the participants usess a number of mechanisms to authenticate responders such as checking for duplicate respondents by evaluating variables such as email address, matches across several demographic data, and device-related data through use of digital fingerprint technology.

b) Participant rights

Participants will be free to withdraw themselves and their data at any point in the research. During the shopping task, clicking on a clearly displayed “exit here” button will lead participants to a quick debrief page that will give them the option to confirm that they do not wish their data to be retained for the study purposes. Participants will be clearly informed before giving consent that anonymity makes withdrawal following completion of the study difficult.

13. FINANCE AND INSURANCE

13.1. Funding

The study is funded by the National Institute for Health Research (NIHR) Oxford Biomedical Research Centre (BRC) and the Collaboration for Leadership in Applied Health Research and Care (CLAHRC Oxford).

13.2. Insurance

This is a simple online task and we cannot foresee any unintended or adverse effects due to participation. The University of Oxford maintains Public Liability and Professional Liability insurance which will operate in this respect.

14. PUBLICATION POLICY

The Investigators will be involved in reviewing drafts of the manuscripts, abstracts, press releases and any other publications arising from the study. Authors will acknowledge that the study was funded by NIHR BRC and CLAHRC. Authorship will be determined in accordance with the ICMJE guidelines and other contributors will be acknowledged. As we will not store the participants' contact details, we will not be able to disseminate the results directly to study participants. We will follow our dissemination plan to engage with public health and health care agencies, the industry, the media, and the public.

15. REFERENCES

1. Berneis KK, Krauss RM. Metabolic origins and clinical significance of LDL heterogeneity. J Lipid Res. 2002;43(9):1363-79.
2. Hooper L, Martin N, Abdelhamid A, Davey Smith G. Reduction in saturated fat intake for cardiovascular disease. Cochrane Database Syst Rev. 2015(6):CD011737.
3. Vafeiadou K, Weech M, Altowaijri H, Todd S, Yaqoob P, Jackson KG, et al. Replacement of saturated with unsaturated fats had no impact on vascular function but beneficial effects on lipid biomarkers, E-selectin, and blood pressure: results from the randomized, controlled Dietary Intervention and VAScular function (DIVAS) study. Am J Clin Nutr. 2015;102(1):40-8.
4. Reidlinger DP, Darzi J, Hall WL, Seed PT, Chowienczyk PJ, Sanders TA, et al. How effective are current dietary guidelines for cardiovascular disease prevention in healthy middle-aged and older men and women? A randomized controlled trial. Am J Clin Nutr. 2015;101(5):922-30.
5. Rees K, Dyakova M, Wilson N, Ward K, Thorogood M, Brunner E. Dietary advice for reducing cardiovascular risk. Cochrane Database Syst Rev. 2013(12):CD002128.
6. Lin JS, O'Connor E, Whitlock EP, Beil TL. Behavioral counseling to promote physical activity and a healthful diet to prevent cardiovascular disease in adults: a systematic review for the U.S. Preventive Services Task Force. Ann Intern Med. 2010;153(11):736-50.
7. Kulick D, Langer RD, Ashley JM, Gans KM, Schlauch K, Feller C. Live well: a practical and effective low-intensity dietary counseling intervention for use in primary care patients with dyslipidemia--a randomized controlled pilot trial. BMC Fam Pract. 2013;14:59.
8. Adams J, Mytton O, White M, Monsivais P. Why Are Some Population Interventions for Diet and Obesity More Equitable and Effective Than Others? The Role of Individual Agency. PLoS Med. 2016;13(4):e1001990.
9. Atkins L, Michie S. Designing interventions to change eating behaviours. Proc Nutr Soc. 2015;74(2):164-70.
10. Broekhuizen K, Kroeze W, van Poppel MN, Oenema A, Brug J. A systematic review of randomized controlled trials on the effectiveness of computer-tailored physical activity and dietary behavior promotion programs: an update. Ann Behav Med. 2012;44(2):259-86.
11. Huang A, Barzi F, Huxley R, Denyer G, Rohrlach B, Jayne K, et al. The effects on saturated fat purchases of providing internet shoppers with purchase- specific dietary advice: a randomised trial. PLoS Clin Trials. 2006;1(5):e22.
12. Hollands GJ, Shemilt I, Marteau TM, Jebb SA, Kelly MP, Nakamura R, et al. Altering micro-environments to change population health behaviour: towards an evidence base for choice architecture interventions. BMC Public Health. 2013;13:1218.
13. Marteau TM, Ogilvie D, Roland M, Suhrcke M, Kelly MP. Judging nudging: can nudging improve population health? Bmj. 2011;342:d228.
14. PlanetRetail. Planet Retail's Online Shopper Survey Data 2014 [Available from: http://www.planetretail.net/presentations/ApexBrasilPresentation.pdf.
15. IGD. The Institute of Grocery Distribution 2017 [Available from: https://www.igd.com/Research/Shopper-Insight/shopper-outlook/13229/Online-grocery-shoppers/.
16. Forwood SE, Ahern AL, Marteau TM, Jebb SA. Offering within-category food swaps to reduce energy density of food purchases: a study using an experimental online supermarket. Int J Behav Nutr Phys Act. 2015;12:85.

16. APPENDIX A: STUDY FLOW CHART

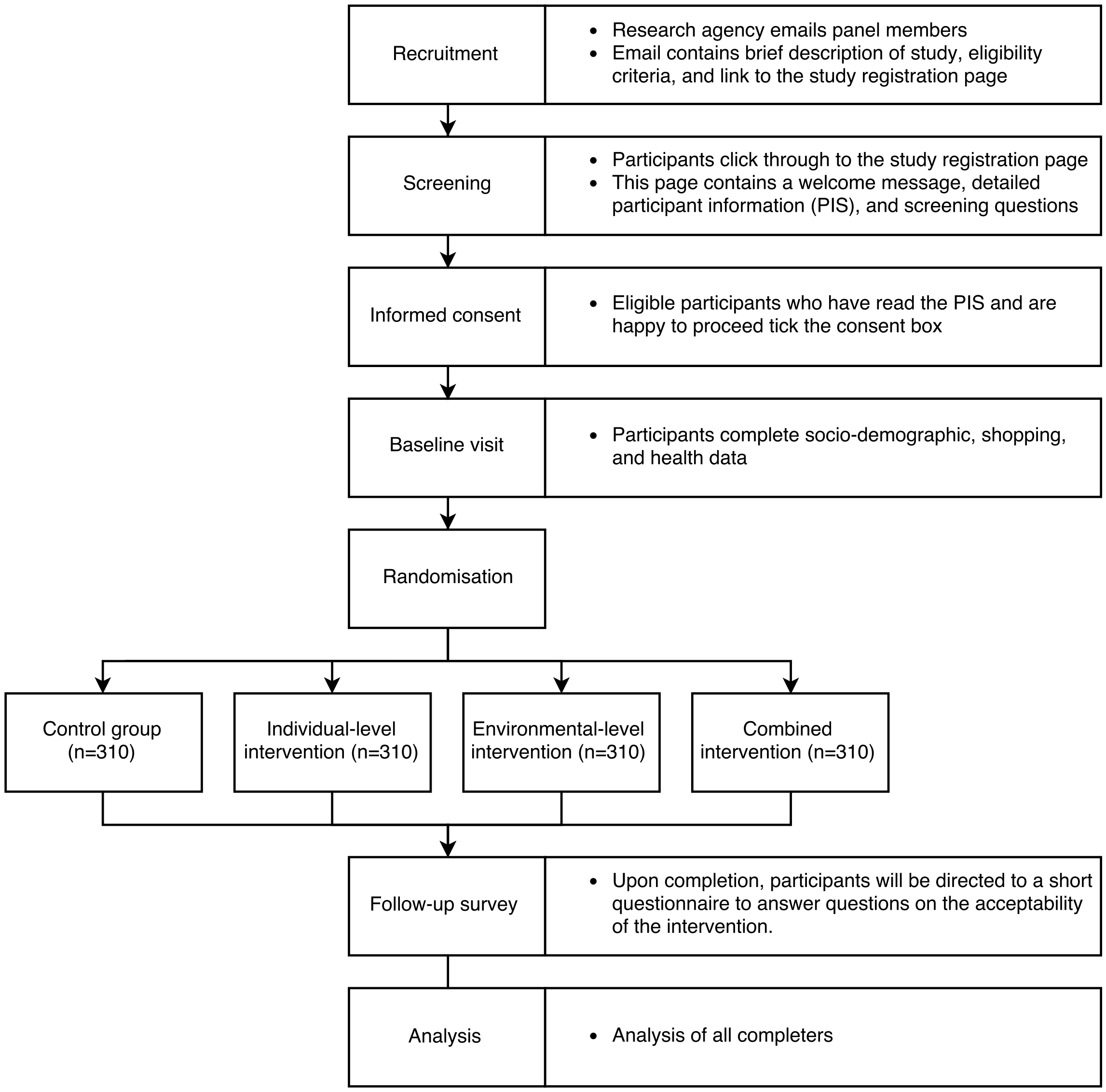

17. APPENDIX B: SCHEDULE OF STUDY PROCEDURES

RECRUITMENT TEXT

The content of the recruitment text will be consistent with other emails sent to panel members but will contain the following text:

Survey topic: Health, Survey length: 30 minutes, Reward: [£5 *[for Prolific participants] OR* xx points *[for ResearchNow participants]].*

Hi [Name],

We have a new survey available for you. By qualifying and completing this task, you will receive [£5 *[for Prolific participants] OR* xx points *[for ResearchNow participants]].*

This study is being carried out by the University of Oxford and may therefore look a little different to surveys you've taken part in before. Rest assured, you will not be asked for any information that could identify you.

This study aims to investigate if two different ways of making healthier choices when shopping online are acceptable to shoppers and effective in reducing the saturated fat in the foods in their basket.

Before undertaking the task, you will be asked to read some detailed participant information, to confirm you are eligible, and confirm your consent to participate.

For further information and to take part go to [link to survey page (PIS and consent) and shopping website].

SCREENING QUESTIONS (Pre-shopping)

1. Age
   - Under 18 years old
   - 18 years old or over

2. Do you currently reside in the UK?
   - Yes
   - No

3. Are you fluent in English?
   - Yes
   - No

4. Do you have any dietary restrictions?
   - Vegetarian
   - Vegan
   - Gluten-free
   - Sugar-free
   - Diary/lactose-free

- Milk allergy
- Eggs allergy
- Nut allergy
- Soy allergy
- Wheat or grain allergy
- Fish allergy
- Shellfish allergy
- Other food allergies
- Other dietary restriction
- None
- Rather not say

5. Are you the main (or shared) grocery shopper for the food that your household eats?
   - Yes
   - No

BASELINE MEASURES (Socio-demographic, shopping and health measures)

1. Demographic characteristics
   - Gender
     - Male
     - Female
     - Other
     - Prefer not to say
   - Age (years)
     - Free text (range of 18-99)
     - Prefer not to say
   - Ethnicity (UK Sensus simplified)
     - White
     - Black
     - Asian
     - Mixed
     - Other
     - Prefer not to say
   - Weight and height (in units chosen by participant).

2. Household income (total household income before tax)

   - Below £15.5K
   - Between £15.5K up to and including £25K
   - Between £25K and £39K
   - £40K or above

3. Household size – "How many people live at your house, including you?

4. Socio-economic status (highest educational level) - "What is the highest education qualification you have achieved?" (categories based on UK census categories)

- None
- Up to 4 GCSE's (Including 1-4 O Levels/CSE/GCSEs (any grades), Foundation Diploma, NVQ level 1, Foundation GNVQ or equivalents)
- 5 or more GCSE's or 1 A-level (Including 5+ GCSEs (Grades A*-C),1 A Level/ 2-3 AS Levels, NVQ level 2, Intermediate GNVQ, City and Guilds Craft, BTEC First/General Diploma, RSA Diploma, Apprenticeship or equivalents)
- 2 or more A-levels (Including 2+ A Levels, 4+ AS Levels, NVQ Level 3, Advanced GNVQ, City and Guilds Advanced Craft, ONC, OND, BTEC National, RSA Advanced Diploma or equivalents)
- Bachelor's degree (Including BA, BSc, NVQ Level 4-5, HNC, HND, RSA Higher Diploma, BTEC Higher level or equivalents)
- Post-Graduate degree or qualification (Including Higher Degrees e.g. MA, PhD, PGCE, Professional qualifications e.g. teaching, nursing, accountancy or equivalents)

5. Regular shopping

"On average, how much do you spend on supermarket shopping per week?" (answer in £)

6. Online shopping experience

"How often, on average over the past year, have you shopped online for food or groceries to be delivered to you (e.g. Tesco.com, Ocado.com, mysupermarket.co.uk)?"

- Never or not in the last year
- 1-3 times in the last year
- 4-11 times in the last year
- 1-3 per month
- once per week or more often.

"How often, on average over the past year, have you shopped online for any non-food items to be delivered to you (e.g. books, clothes, electronics)?"

- Never or not in the last year
- 1-3 times in the last year
- 4-11 times in the last year
- 1-3 per month
- once per week or more often.

7. Health related questions

"Have you ever been advised by your doctor or health professional to reduce the amount of saturated fat in your diet?"

- Yes/No

"Have you ever been diagnosed with one of the following health conditions?"

- Heart disease
- High cholesterol
- High blood pressure
- Diabetes
- Obesity or overweight
- Cancer
- COPD (Chronic Obstructive Pulmonary Disease)
- None of the above

POST-SHOPPING TASK PARTICIPANT SURVEY

1. "The online supermarket you have just used may have offered you alternative products which contained less saturated fat than the foods you originally chose. Were you offered any swaps in this shopping task today?"

- "Yes/No"

1A. "Is this a feature you would like to have when you do your usual shopping?"

- Strongly agree
- Somewhat agree
- Indifferent
- Somewhat disagree
- Strongly disagree

2. "When making a choice of foods or drinks to buy, what are the top 3 things that affect your decision?"

- Price
- Appearance
- Taste (preference)
- Habits
- Healthiness
- Convenience (to prepare or to consume)
- Special offers
- Organic
- Special diet e.g. gluten free, nut free
- Other (e.g. animal welfare, locally produced, packaging)

3. "How often do you look at the nutrition labels for the following, when doing your usual grocery shop?"

- Calories: Always/Often/Sometimes/Rarely/Never
- Fat: Always/Often/Sometimes/Rarely/Never
- Saturated fat: Always/Often/Sometimes/Rarely/Never
- Sugar: Always/Often/Sometimes/Rarely/Never
- Salt: Always/Often/Sometimes/Rarely/Never

4. "Is there anything else you'd like to tell us about your experience with this shopping task today?" (Please do NOT include your name here).

[FREE TEXT ANSWER]

18. APPENDIX C: AMENDMENT HISTORY

Amendment No.	Protocol Version No.	Date issued	Author(s) of changes	Details of Changes made
1	2.0	28-Mar-2018	Dimitrios Koutoukidis	Update of the sample size calculation
2	3.0	13-Jul-2018	Dimitrios Koutoukidis	Change in the definition of the primary objective and outcome by deleting the word "change". This was an inaccuracy, as change cannot be calculated in this trial.<br><br>Inclusion of a comparison of the two single interventions in the primary outcome. This was originally intended and it is produced as standard output from the statistical analysis (ANOVA).<br><br>Reordering of the secondary objectives (and respective outcome measures) to aid clarity. Not all objectives can be met by comparisons between each arm. The objectives that refer to comparisons between each arm have been grouped together. The same applied to the comparisons between the individual-level and combined interventions. We have split the objective "the proportion of swaps accepted out of those offered" into three objectives to aid clarity. The same applied to the respective outcome measures.<br><br>Reference to the detailed pre-planned statistical analysis in the SAP.

STATISTICAL ANALYSIS PLAN

The OLS Study

The effectiveness of individual-level and environmental-level interventions on food choices:

an experimental online supermarket study

Version number and date: 1.0, 13-Jul-2018

Version History

Version:	Version Date:	Changes:

Table of Contents

1 Introduction 30

1.1 Preface 30

1.2 Purpose and scope of the plan 30

1.3 Trial overview 30

1.4 Objectives 30

2 Trial design 31

2.1 Outcomes measures 31

2.1.1 Primary outcome 31

2.1.2 Secondary outcomes 31

2.2 Target population 32

2.3 Sample size 32

2.4 Randomisation and blinding in the analysis stage 32

3 Analysis – General considerations 34

3.1 Descriptive statistics 34

3.2 Characteristics of participants 34

3.3 Definition of population for analysis 34

3.4 Data Monitoring Committee And Interim Analyses 34

4 PRIMARY ANALYSIS 34

4.1 Primary outcome 34

4.2 Handling missing data 35

4.3 Handling outliers 35

4.4 Multiple comparisons and multiplicity 35

4.5 Model assumptions 35

5 SECONDARY ANALYSIS 36

5.1 Secondary outcomes 36

5.2 Non-efficacy outcomes 36

6 SUBGROUP ANALYSES 36

7 ADDITIONAL EXPLORATORY ANALYSIS 36

8 SAFETY ANALYSIS 36

8.1 Adverse events 37

9 VALIDATION 37

10 CHANGES TO THE PROTOCOL OR PREVIOUS VERSIONS OF SAP 37

11 Appendices 38

1 Introduction

1.1 Preface

The Trial Statistician (Dr Jose Ordoñez-Mena), Chief Investigator (Dr Carmen Piernas-Sanchez), and Trial Manager/Co-Investigator (Dr Dimitrios Koutoukidis) have contributed to and approved the statistical analysis plan (SAP). The SAP supports the study protocol version 2.0 and dated 05-Feb-2018. Analysis will be carried out using up-to-date versions of Microsoft Word and R.

1.2 Purpose and scope of the plan

The purpose of the plan is to complete the main analysis as stated in the protocol.

1.3 Trial overview

High saturated fat intake increases the risk of cardiovascular disease. Dietary counselling has proven to have only modest effects. An approach that also includes environmental restructuring may reduce the risk of widening inequalities and may be more successful in sustaining individual behaviour change. Food purchasing is a key antecedent of food consumption, therefore individual-level interventions targeting the nutritional quality of the grocery shopping present a clear opportunity for action. Online supermarkets offer unique opportunities to deliver and support complex nutrition interventions to shape food purchasing patterns at scale, but research in this arena is still in its infancy and more solid evidence is required to develop a truly effective intervention with population-level impact.

The aim of this project is to test the effects of an individual-level intervention and an environmental-level intervention, separately and in combination, on SFA in a sample of UK adults.

1.4 Objectives

Primary objective

To investigate the magnitude of saturated fat achieved in the shopping basket in response to an individual-level intervention and an environmental-level intervention, separately and in combination, compared to control (no intervention).

Secondary objectives

To investigate the effect of the interventions on:

i. the proportion of products with less than 1.5% saturated fat in the final basket
ii. the overall cost of the final shopping basket
iii. the diet composition of the shopping basket

To investigate the effect of the individual-level intervention on

iv. saturated fat change per swap accepted
v. the proportion of swaps accepted out of those offered overall
vi. the proportion of swaps accepted out of those offered by magnitude of reduction
vii. the proportion of swaps accepted out of those offered by type of food group
viii. the proportion of accepted swaps out of total shopping basket items

2 Trial design

This is a 2x2 factorial randomised controlled trial of an individual-level and an environmental-level intervention to reduce the SFA content of the total shopping basket during an online shopping experiment. Each participant will be randomly allocated on an 1:1:1:1 basis to one of the four trial arms and participate in the study for about 30 minutes in total (see Appendix A of the study protocol for a study flow chart). The interventions are detailed on section 7 of the study protocol. UK adults with no dietary restrictions who are the main grocery shoppers for their household will be invited to participate through an online research agency (https://www.prolific.ac/).

Following consent, participants will complete a baseline questionnaire on demographic, shopping, and health data. Participants will complete a short post-intervention survey about the acceptability of the two interventions in the online shopping task and their usual shopping behaviours.

The investigators and the participants will not be blinded. The outcome assessor and the trial statistician will be blinded.

2.1 Outcomes measures

2.1.1 Primary outcome

As primary outcome, we will compare the difference in the saturated fat content of the final basket (measured in % of total energy) between each of the four trial arms.

2.1.2 Secondary outcomes

The following outcomes will be compared between each of the four trial arms:

i. difference in the proportion of products with lower saturated fat in the final basket (%)
ii. difference in the overall cost of the final shopping basket (£) weighted for the size of the basket (g)
iii. difference in the total energy, energy density, sugars (% energy), and salt (g/100g) content of the shopping basket

The following outcomes will be compared between the single individual-level intervention (swaps only) and the combined intervention arms

iv. difference in % saturated fat content per swap accepted (% energy intake)
v. difference in the proportion of swaps accepted out of those offered (%)
vi. difference in the proportion of swaps accepted out of those offered (%) by median observed change in saturated fat
vii. difference in the proportion of swaps accepted out of those offered (%) for (a) butter, margarine, and spreads, (b) cheese, (c) milk, (d) meat, and (e) sweets and desserts
viii. difference in the proportion of accepted swaps out of total shopping basket items (%)

Non-efficacy outcome:

Rating scores from the survey on acceptability and open-ended answers from the follow-up questionnaires

2.2 Target population

Inclusion Criteria

- UK adults, aged ≥18 years.
- Able to speak and read English.
- Willing and able to give informed consent for participation in the study.
- Being the main (or shared) grocery shopper for their household.
- Having access to a computer and Internet.

Exclusion Criteria

- Having any dietary restriction.

2.3 Sample size

There are no previously reported standard deviations of the mean difference from similar trials to guide the estimation of the standard deviation in our trials, and, thus, for the calculation of the sample size.

Therefore, we conducted an initial pilot to estimate the standard deviation and adapt the power calculation if necessary. The initial plan was to recruit 500 people for the pilot and include those in the analysis of the full trial. Due to logistical constraints, 129 people were recruited for an independent pilot. This sample included 31-33 participants per arm with valid data, as our pre-defined completion criterion (i.e. people who bought at least 5 items from the list), and showed a standard deviation of 6.5%.

If we were to observe a minimally clinically relevant 2% reduction in SFA (assuming a 7% - rounded 6.5 - standard deviation) in the total basket between any of the 4 groups using intention to treat analyses with 90% power and two-sided $\alpha=0.05$, we would require 258 per group giving a total of 1032 participants. A final sample of 1240 participants would account for 20% non-completion through participants not completing the shopping task.

2.4 Randomisation and blinding in the analysis stage

The statistician generated the randomisation sequence in R (see Appendix 1 for R code) and the investigator uploaded the sequence to the survey platform (https://redcap.phc.ox.ac.uk/). Randomisation was performed by the survey platform via computerised random number generation on an 1:1:1:1 basis with random block sizes and participants were directed to a website that introduces an online shopping task. Allocation concealment was achieved, as participants were

recruited from independent research panels and were directed for automatic randomisation in the survey platform.

Investigators were not blinded to intervention allocation but they were not able to manipulate any study parameter following the initial study set up, as all study procedures are taking part in the online platform. The outcome assessment is blinded, as it happens automatically in the online platform. The statistician who will analyse the data will be blinded to intervention allocation. Due to the nature of the intervention, it will not be possible to blind participants to the intervention. However, participants will only be aware of the trial arm that they are exposed to and will be unaware of the other trial arms.

3 Analysis – General considerations

3.1 Descriptive statistics

A table will present the baseline characteristics by trial arm and overall (Appendix 2). The table will include age, gender, ethnic group, weight, BMI, education, household income, household size, household supermarket spending, online shopping, and health conditions. Continuous variables will be summarised using means and standard deviations. Medians with interquartile ranges will be presented where appropriate. Categorical variables will be summarised using counts and percentages. Data will be analysed using R.

3.2 Characteristics of participants

Baseline characteristics will include age, gender, ethnic group (White, Black, Asian, Mixed, Other), BMI (continuous and categories based on the WHO cut-offs), education (none, secondary education, higher education), household income (<£15.5k, 15.5-25k, 26-39, ≥40), household size (continuous in number of people), household supermarket spending (continuous in £), online shopping for groceries (Once per week or more often, 1-3 times per month, 4-11 times in the last year, 1-3 times in the last year, Never or not in the last year), online shopping for non-food items (Once per week or more often, 1-3 times per month, 4-11 times in the last year, 1-3 times in the last year, Never or not in the last year) and history of health conditions (heart disease, high cholesterol, high blood pressure, diabetes, obesity or overweight, COPD, none of the above).

3.3 Definition of population for analysis

We will use an intention-to-treat approach (based on the trial arm participants were initially randomised) to analyse all participants who completed the shopping task (available case analysis). Completion of the shopping task will be defined as purchase of at least 5 out of 10 items from the categories listed in section 7 of the study protocol. The sample size calculation has accounted for a 20% non-response rate. We do not expect major protocol violations, such as violation of entry criteria, due to the online computerised nature of the trial management and delivery.

3.4 Data Monitoring Committee And Interim Analyses

Due to the low risk of harm and short length of the intervention, a data monitoring committee will not be needed and an interim analysis will not be conducted.

4 PRIMARY ANALYSIS

4.1 Primary outcome

The null hypothesis is that this is no effect of the interventions and the two-sided alternative hypothesis is that there is a difference in saturated fat measured in % of total energy between any of the 4 trial arms. The observed difference will be interpreted in light of a 2% reduction in saturated fat intake which is deemed minimally clinically relevant. The sum of saturated fat (g) will be multiplied by 9 and then divided by the sum of the energy (kcal) in each participant's final basket.

Two-way analysis of variance (ANOVA) will be used to test for the difference between

- SFA in each intervention group compared to control
- SFA in the combined intervention group compared to single intervention groups
- SFA in one single intervention group versus the other single intervention group
- SFA in the combined intervention group compared to control.

Estimates of intervention effects will be reported with confidence intervals.

4.2 Handling missing data

The percentage and absolute withdrawal in each study arm will be reported in the CONSORT flow-chart and reasons for withdrawal will be documented. As the outcome variables (saturated fat in g and energy in kcal) are automatically calculated based on the food database embedded in the online platform, we do not anticipate any missing for the population for analysis.

4.3 Handling outliers

For the analysis of the primary outcome, we do not expect significant outliers based on our definition of population for analysis (section 3.3). Data outliers will be defined as being at least three standard deviations from the mean of its distribution in the variable at that time-point and will be cross-checked. Given calculation of the primary outcome using the pre-existing nutrition database, we do not expect significant outliers in the primary outcome measure. Outliers will be included in the analysis and a sensitivity analysis will be conducted by setting outliers to be missing.

4.4 Multiple comparisons and multiplicity

As the comparisons have been pre-specified, we will not correct for multiple testing.

4.5 Model assumptions

The appropriateness of the normality, no outliers, and homogeneity of variances assumptions required for the ANOVA model will be assessed using residual and other diagnostic plots, the Shapiro-Wilk test of normality, and the Levene's test for equality of variances. Where concern is indicated, a transformation and/or a nonparametric method will be used to address gross deviations from the assumptions. It is unlikely that the primary outcome will need to be transformed in order to make use of methods assuming normality.

5 SECONDARY ANALYSIS

5.1 Secondary outcomes

Two-way ANOVA will be used to test for the difference between each trial arm

- in the proportion of products with lower saturated fat in the final basket (%)
- difference in the overall cost of the final shopping basket (£) weighted for the size of the basket in grams
- difference in the total energy, energy density (kcal/g), sugars (% energy) and salt (g/100g) content of the shopping basket

Independent t-test will be used for the difference between the single individual-level intervention (swaps only) and the combined intervention arms

- difference in % saturated fat content per swap accepted (% energy intake)
- difference in % swaps accepted out of those offered (%)
- difference in % swaps accepted out of those offered (%) by median observed change in saturated fat
- difference in % swaps accepted out of those offered (%) for (a) butter, margarine, and spreads, (b) cheese, (c) milk, (d) meat, and (e) sweets and desserts
- difference in % swaps accepted out of the total number of products in the basket

5.2 Non-efficacy outcomes

The rating scores of the acceptability of swaps will be presented descriptively.

The open-ended follow-up questionnaire will be analysed using content analysis in MS Excel.

6 SUBGROUP ANALYSES

We do not expect the intervention effect to differ between subgroups. However, we will conduct exploratory analysis by gender, age group stratified by median, ethnic group (White vs Non-White), BMI stratified into <30 and ≥30kg/m^2 groups, highest education level (lower vs. higher), and household income (lower vs. higher) provided we have sufficient numbers within each subgroup (n≥30). We will also run an analysis by the purchased food group.

7 ADDITIONAL EXPLORATORY ANALYSIS

We have not planned any additional exploratory analysis.

8 SAFETY ANALYSIS

Due to the low risk of harm, there is no plan for a safety analysis.

8.1 Adverse events

This is a simple online task and we cannot foresee any unintended or adverse effects due to participation.

9 VALIDATION

A senior statistician will double check the analysis plan and code, and re-run the code for the primary analysis.

10 CHANGES TO THE PROTOCOL OR PREVIOUS VERSIONS OF SAP

N/A

11 Appendices

Appendix I. Randomisation code in R

```
install.packages("blockrand")
library(blockrand)

set.seed(123)

rand <- blockrand(n=1240, levels = c("26","27","28","29"))

table(rand$treatment)

table(rand[1:1240,"treatment"])

save.folder <- '/Users/dimitrioskoutoukidis/Desktop/'

write.csv(rand[,c("id","treatment")], row.names = FALSE,
          paste0(save.folder, 'OLS Random Allocation.csv'))
```

Appendix 2: Template tables for presentation of results

Baseline characteristics of participants

N(%), unless otherwise specified	Control (n=)	Swaps (n=)	Positioning (n=)	Combination (n=)
Age, years, mean (SD)				
Gender, female				
BMI, kg/m^2, mean (SD)				
BMI categories				
Underweight (<18.5)				
Normal weight (18.5-24.9)				
Overweight (25-29.9)				
Obesity (≥30)				
Ethnic group				
White				
Black / Asian				
Mixed / Other				
Education				
No formal qualifications				
Secondary education				
Higher education				
Household income				
Lower (≤£25k)				
Middle (£26-39k)				
Higher (≥£40k)				
Household size, median (IQR)				
Household supermarket spending, median (IQR)				
Online grocery shopping				
≥1 per week				
1-3 times per month				
4-11 times in the last year				
1-3 times in the last year				
Never or not in the last year				
Online non-grocery shopping				
≥1 per week				
1-3 times per month				
4-11 times in the last year				
1-3 times in the last year				

Never or not in the last year				
Health history				
Heart disease				
High cholesterol				
High blood pressure				
Diabetes				
Overweight/obesity				
COPD				

Primary and secondary outcomes between trial arms, all mean± SD

	Mean± SD				Between group difference (95% CI)					
	Control (n=)	Swaps (n=)	Positioning (n=)	Combination (n=)	Swaps vs Control	Positioning vs Control	Combination vs Control	Combination vs Swaps	Combination vs Positioning	Positioning vs Swaps
Primary outcome										
SFA (% kcal)										
Secondary outcomes										
% of products with <1.5% SFA										
Cost (£/100g)										
Total energy (kcal)										
Energy density (kcal/g)										
Sugar (% kcal)										
Salt (g/100g)										

Outcomes between swaps and combination arms

	Swaps		Combination		Combination vs Swaps
	Mean± SD	n	Mean± SD	n	Between group difference (95% CI)
SFA (% kcal) per accepted swap					
% swaps accepted out of swaps offered					
total					
High SFA change					
Low SFA change					
Butter, margarine, spreads					
Cheese					
Milk					
Meat					
Sweets and desserts					
% of accepted swaps out of total number of basket items					

Subgroup analysis

	Mean± SD, N								Between group difference (95% CI)					
SFA (% kcal)	Control		Swaps		Positioning		Combination		Swaps vs Control	Positioning vs Control	Combination vs Control	Combination vs Swaps	Combination vs Positioning	Positioning vs Swaps
Sex														
Female														
Male														
Age														
Median and above														
Below median														
Ethnic group														
White														
Non-White														
BMI														
<30														
>30														
Education														
Lower														
Higher														
Household income														
Lower														
Higher														

Main effects and interactions

	Mean± SD				Between group difference (95% CI)		Interaction coefficient (95% CI)
	Swaps (n=)	No Swaps (n=)	Positioning (n=)	No Positioning (n=)	Swaps vs Control	Positioning vs Control	
Primary outcome							
SFA (% kcal)							
Secondary outcomes							
% of products with <1.5% SFA							
Cost (£/100g)							
Total energy (kcal)							
Energy density (kcal/g)							
Sugar (% kcal)							
Salt (g/100g)							

Post-task survey on 3 most important factors affecting food purchasing decisions (Yes/No)

N(%)	Control (n=)	Swaps (n=)	Positioning (n=)	Combination (n=)	Total (n=)
Price					
Appearance					
Taste					
Habits					
Healthiness					
Convenience					
Special offers					
Organic					
Special diet (e.g. gluten free)					
Other (e.g. animal welfare, locally produced, packaging))					

Post-task survey on looking at nutrition labelling while at usual grocery shopping (total n=)

N(%)	Always	Often	Sometimes	Rarely	Never
Calories					
Fat					
Saturated fat					
Sugar					
Salt					
