# Supplementary material for: Prominent positioning and food swaps are effective interventions to reduce the saturated fat content of the shopping basket in an experimental online supermarket: a randomized controlled trial
Source: Int J Behav Nutr Phys Act. 2019 Jun 7;16:50. doi: 10.1186/s12966-019-0810-9 (PMC6555993; doi:10.1186/s12966-019-0810-9)
Supplement: Supplementary file 2 — Supplementary figures and tables (Description of data: Examples of the interventions and additional analysis). (PDF 78 kb) [file 12966_2019_810_MOESM2_ESM.pdf]

Additional file 2: Supplementary tables

Table S1 Time spent on the supermarket platform and number of items browsed and bought.

	Mean (SD)				Mean Difference (95% Confidence interval)					
	**Control** (n=276)	**Swaps** (n=279)	**Altering order** (n=261)	**Combined** (n=273)	**Swaps vs Control**	**Altering order vs Control**	**Combined vs Control**	**Altering order vs Swaps**	**Combined vs Swaps**	**Combined vs Altering order**
**Time (min) spent on the supermarket**	14.2 (6.8)	16.4 (8.4)	17.2 (7.9)	17.5 (7.8)	2.2 (0.5 to 3.9)	3.0 (1.3 to 4.7)	3.3 (1.6 to 5.0)	0.8 (-0.9 to 2.5)	1.1 (-0.6 to 2.8)	0.3 (-1.5 to 2.0)
**N shelf and product pages browsed**	25.48 (12.77)	28.08 (15.13)	30.93 (14.96)	30.18 (15.58)	2.6 (-0.6 to 5.8)	5.45 (2.19 to 8.71)	4.7 (1.48 to 7.92)	2.85 (-0.39 to 6.1)	2.1 (-1.11 to 5.31)	-0.76 (-4.02 to 2.51)
**N product pages browsed**[1]	0 (0)	0 (0.5)	0 (1)	0 (0)	-0.02 (-0.54 to 0.49)	0.21 (-0.32 to 0.73)	-0.04 (-0.56 to 0.48)	0.23 (-0.29 to 0.76)	-0.02 (-0.54 to 0.5)	-0.25 (-0.78 to 0.28)
**N products bought**	11.41 (4.16)	12.37 (6.67)	11.26 (3.94)	11.62 (5.75)	0.95 (-0.2 to 2.11)	-0.15 (-1.33 to 1.02)	0.21 (-0.95 to 1.37)	-1.11 (-2.28 to 0.06)	-0.74 (-1.9 to 0.41)	0.37 (-0.81 to 1.54)

[1]These data are presented as median (interquartile range) with median differences between groups.

Table S2 Main effects and test for interaction

	Swaps (n=552)	No Swaps (n=536)	Altering order (n=534)	No Altering order (n=554)	Swaps: Yes vs No	Altering: Yes vs No	P Interaction
**Primary outcome**							
SFA (% kcal)	22 (6.47)	23.25 (6.46)	20.48 (6.37)	24.67 (5.92)	-1.25 (-2.02 to -0.48)	-4.2 (-4.93 to -3.47)	0.04
**Secondary outcomes**							
% of products with <1.5% SFA	28.35 (15.37)	25.5 (15.23)	31.65 (16.05)	22.41 (13.18)	2.85 (1.03 to 4.67)	9.25 (7.5 to 10.99)	0.17
Cost (£/100g)	0.37 (0.11)	0.36 (0.11)	0.36 (0.11)	0.36 (0.12)	0.01 (-0.01 to 0.02)	0 (-0.02 to 0.01)	0.77
Total energy (kcal)	13541 (7793)	13484 (6578)	12562 (6799)	14430 (7490)	57 (-802 to 916)	-1868 (-2720 to -1016)	0.73
Energy density (kcal/g)	201.05 (46.37)	207.2 (48.28)	192.7 (45.15)	215.05 (46.97)	-6.14 (-11.77 to -0.51)	-22.35 (-27.83 to -16.86)	0.48
Sugar (% energy)	16.59 (5.53)	16.51 (5.86)	17.74 (6.16)	15.41 (4.95)	0.08 (-0.6 to 0.76)	2.33 (1.67 to 3)	0.77
Salt (g/100g)	0.15 (0.07)	0.15 (0.1)	0.14 (0.06)	0.15 (0.11)	-0.01 (-0.02 to 0.01)	-0.01 (-0.02 to 0)	0.46

The first four columns include mean (SD), the next 2 columns show mean difference and 95% CIs.

Table S3 Change in % energy from saturated fat of the basket by number of swaps accepted (Table based on Figure 3)

	Swaps alone		Combined		Median difference (95% CI)
	**Median (IQR)**	**n**	**Median (IQR)**	**n**	
**SFA (% kcal) per accepted swap**					
**Accepted 0 swap**					
Before	24.3 (20.9 - 27.6)	103	18.1 (13.6 - 22.57)	164	6.24 (4.63 to 7.67)
After	24.3 (20.9 - 27.6)	103	18.1 (13.6 - 22.57)	164	6.24 (4.66 to 7.72)
Difference	0.0 (0 - 0)	103	0.0 (0 - 0)	164	0.00 (0 to 0)
**Accepted 1 swap**					
Before	23.5 (20.2 - 26.44)	71	19.6 (16.7 - 22.8)	77	3.95 (1.86 to 5.38)
After	23.1 (19.1 - 25.63)	71	18.8 (15.3 - 21.95)	77	4.30 (1.82 to 5.27)
Difference	0.8 (0.4 - 1.38)	71	0.7 (0.3 - 1.36)	77	0.04 (-0.17 to 0.31)
**Accepted 2+ swaps**					
Before	24.4 (21 - 26.79)	105	19.5 (16.4 - 24.18)	32	4.85 (1.57 to 5.81)
After	21.6 (19.1 - 24.6)	105	18.7 (13.7 - 22.45)	32	2.92 (1.11 to 5.4)
Difference	1.8 (1.1 - 2.98)	105	1.8 (1.1 - 2.63)	32	-0.01 (-0.42 to 0.69)

In the combination group, only 148 were offered at least 1 swap out of the 164 that accepted 0 swaps (i.e. 16 people were not offered any swap).

Table S4 Subgroup analysis between intervention groups

	Between group mean difference (95% confidence intervals)		
	**Swaps vs Control**	**Altering order vs Control**	**Combined vs Control**
**Sex**			
Female	-1.28 (-2.92 to 0.35)	-4.32 (-5.97 to -2.68)	-4.47 (-6.12 to -2.81)
Male	-3.27 (-5.56 to -0.99)	-6.22 (-8.62 to -3.82)	-7.09 (-9.37 to -4.81)
**Age**			
Median and above	-2.84 (-4.77 to -0.91)	-4.21 (-6.17 to -2.24)	-4.71 (-6.69 to -2.74)
Below median	-1.05 (-2.88 to 0.79)	-5.77 (-7.63 to -3.92)	-6.02 (-7.83 to -4.22)
**Ethnic group**			
White	-1.89 (-3.3 to -0.48)	-4.85 (-6.28 to -3.42)	-5.44 (-6.87 to -4.02)
Non-White	-2.8 (-7.38 to 1.78)	-6.3 (-11.1 to -1.5)	-5.09 (-9.36 to -0.82)
**BMI**			
<30	-2.14 (-3.72 to -0.56)	-5.75 (-7.39 to -4.12)	-5.22 (-6.79 to -3.65)
>30	-1.83 (-4.33 to 0.68)	-3.32 (-5.81 to -0.84)	-6.05 (-8.67 to -3.43)
**Education**			
None/Secondary	-1.57 (-4.09 to 0.95)	-5.03 (-7.57 to -2.49)	-5.2 (-7.86 to -2.53)
Higher	-2.06 (-3.67 to -0.46)	-4.76 (-6.4 to -3.12)	-5.45 (-7.01 to -3.88)
**Household income**			
Low-middle (£25-39k)	-2.21 (-3.82 to -0.59)	-5.06 (-6.69 to -3.43)	-5.61 (-7.25 to -3.97)
Higher (£40k+)	-1.34 (-3.8 to 1.13)	-4.53 (-7.11 to -1.96)	-4.6 (-7.05 to -2.15)

Table S5 Post-task survey on the most important factors affecting choice of purchase of foods or drinks by trial arm (n, %).

When making a choice of foods or drinks to buy, what are the top 3 things that affect your decision?										
**N %**	**Control**		**Swaps**		**Altering order**		**Both**		**Total**	
Price	216	70	252	81	207	67	232	75	907	73
Taste[1]	209	67	233	75	217	70	214	69	873	70
Healthiness	70	23	99	32	84	27	93	30	346	28
Special offers	86	28	84	27	54	17	101	33	325	26
Habits	87	28	61	20	82	26	59	19	289	23
Convenience	86	28	52	17	72	23	63	20	273	22
Appearance	54	17	37	12	43	14	39	13	173	14
Organic	8	3	4	1	7	2	7	2	26	2
Special diet[1]	1	0	5	2	4	1	1	0	11	1
Other[2]	13	4	8	3	12	4	16	5	49	4

[1] Theirs or their families', [2] e.g. animal welfare, locally produced, packaging.

Table S6 Post-task survey on the frequency of checking the nutrition labelling while at usual grocery shopping

How often do you look at the nutrition labels for the following, when doing your usual grocery shop?						
**N (%)**	**Never**	**Rarely**	**Sometimes**	**Often**	**Always**	**Missing**
Calories	142 (13.1)	219 (20.1)	356 (32.7)	271 (24.9)	91 (8.4)	9 (0.8)
Fat	135 (12.4)	258 (23.7)	382 (35.1)	240 (22.1)	64 (5.9)	9 (0.8)
Saturated fat	180 (16.5)	303 (27.8)	333 (30.6)	202 (18.6)	61 (5.6)	9 (0.8)
Sugar	120 (11)	212 (19.5)	342 (31.4)	302 (27.8)	103 (9.5)	9 (0.8)
Salt	180 (16.5)	365 (33.5)	292 (26.8)	183 (16.8)	59 (5.4)	9 (0.8)
